# Design, Synthesis, and Biological Evaluation Studies of Novel Naphthalene-Chalcone Hybrids As Antimicrobial, Anticandidal, Anticancer, and VEGFR-2 Inhibitors

**DOI:** 10.1021/acsomega.2c07256

**Published:** 2023-02-13

**Authors:** Derya Osmaniye, Begüm Nurpelin Sağlık, Narmin Khalilova, Serkan Levent, Gizem Bayazıt, Ülküye Dudu Gül, Yusuf Özkay, Zafer Asım Kaplancıklı

**Affiliations:** † Department of Pharmaceutical Chemistry, Faculty of Pharmacy, Anadolu University, 26470 Eskişehir, Turkey; ‡ Doping and Narcotic Compounds Analysis Laboratory, Faculty of Pharmacy, Anadolu University, 26470 Eskişehir, Turkey; § Vocational School of Health Services, Biotechnology Application and Research Center, Bilecik Seyh Edebali University, 11230 Bilecik, Turkey

## Abstract

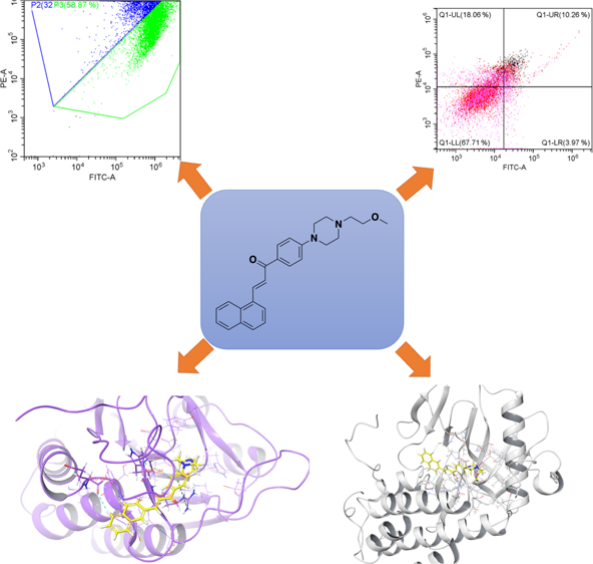

Cancer is a progressive disease that is frequently encountered worldwide. The incidence of cancer is increasing with the changing living conditions around the world. The side-effect profile of existing drugs and the resistance developing in long-term use increase the need for novel drugs. In addition, cancer patients are not resistant to bacterial and fungal infections due to the suppression of the immune system during the treatment. Rather than adding a new antibacterial or antifungal drug to the current treatment plan, the fact that the drug with anticancer activity has these effects (antibacterial and antifungal) will increase the patient’s quality of life. For this purpose, in this study, a series of 10 new naphthalene-chalcone derivatives were synthesized and their anticancer-antibacterial-antifungal properties were investigated. Among the compounds, compound **2j** showed activity against the A549 cell line with an IC_50_ = 7.835 ± 0.598 μM. This compound also has antibacterial and antifungal activity. The apoptotic potential of the compound was measured by flow cytometry and showed apoptotic activity of 14.230%. The compound also showed 58.870% mitochondrial membrane potential. Compound **2j** inhibited VEGFR-2 enzyme with IC_50_ = 0.098 ± 0.005 μM. Molecular docking studies of the compounds were carried out by *in silico* methods against VEGFR-2 and caspase-3 enzymes.

## 1 Introduction

The disorder known as cancer, which can arise from any cell and develop anywhere in the body, is spreading across the world. Cancer is the most serious condition, according to statistics and the growth of cancer cells.^1^ With an estimated 1.8 million deaths (18%), lung cancer remained the most common type of cancer death in 2020, followed by colorectal (9.4%), liver (8.3%), stomach (7.7%), and female breast (6.9%) cancer. Globally, it was estimated that approximately 19.3 million new cancer cases and nearly 10 million cancer deaths occurred in 2020.^2^ The potential of developing cancer is increased by a number of risk factors, including alcohol and cigarette use, exposure to carcinogens, obesity, and family history.^3^ Cancer presents a significant challenge to the medical research community in terms of developing treatments, therapies, and strategies for more efficient care for patients.^4^ Naphthalene ring is a ring rich in biological activity. There are many studies in the literature on the anticancer activity of naphthalene ring.^5^ There are also many studies on the antimicrobial activity of this ring.^6^

Although chalcones are α, β unsaturated ketone compounds in a 1,3-diaryl-2-propenone structure, their importance in medicinal chemistry is increasing due to their easy synthesis and simple chemistry. The synthesis of chalcones is carried out by different reaction types, such as Claisen–Schmidt reactions, Heck coupling, Suzuki–Miyaura coupling, and Witting reactions. They are compounds with very wide biological activity according to their substitutions in the aryl structure. Examples of these are anti-inflammatory, antimicrobial, anticancer, cytotoxic, and anticholinesterase.^7^ In addition, there are many publications showing the anticancer or antimicrobial activities of chalcones.^8^

In cellular activities, such as growth, survival, invasion, and angiogenesis during tumor initiation and development, protein kinases play different signaling pathways.^9^ The initial tumor and any resulting metastases are dependent on angiogenesis, according to recent research. Several protein kinases, including the growth factors, regulate the angiogenesis process. The vascular endothelial growth factor (VEGF) is one of the most potent angiogenic determinants that can control angiogenesis and be involved in the development of a tumor among those growth factors.^10^ By interacting with VEGF receptor types 1–3, the VEGF family controls angiogenesis.^11^ The VEGFR-2 binding site is hydrophobic, hence VEGFR-2 inhibitors showed a wide variety of chemical configurations. However, there were significant similarities between the chemical structures of the two well-known inhibitors (sorafenib I and regorafenib II) that are necessary for any inhibitor to correctly engage with the active binding site.^12^

**Figure 1:**
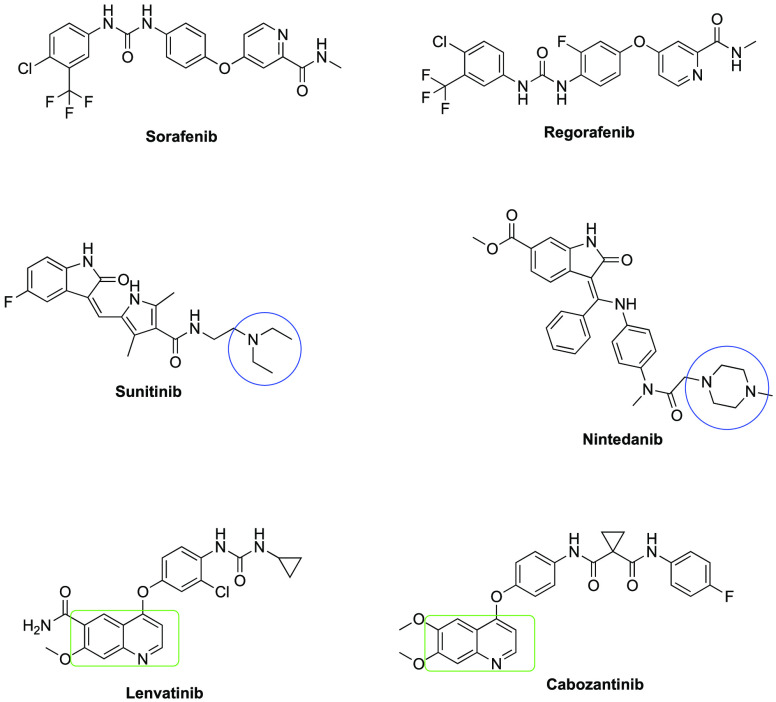
Some VEGFR inhibitors.

In light of the above information, 10 new compounds were synthesized within the scope of this study. The naphthalene ring of the compounds was used as the bioisostere of the quinoxaline ring in the structures of the third generation VEGFR inhibitor Lenvatinib and Cabozantinib compounds (Figure 1). The piperazine ring was preferred because of the secondary amine derivatives in Sunitinib and Nintedanib, which are second generation VEGFR inhibitors.

## 2 Results and Discussion

### 2.1 Chemistry

The compounds **2a**–**2j** were synthesized as presented in Scheme 1. First, the ketone derivatives (**1a**–**1e**) were obtained by using 4-fluoroacetophenone and appropriate secondary amine. Between 1-naphtaldehyde or 2-naphtaldehyde and the ketone derivatives (**1a**–**1e**), Claisen Schmitt reaction was carried out for obtain target compounds (**2a**–**2j**). Spectroscopic techniques, such as ^1^H NMR, ^13^C NMR, and HRMS, were used to demonstrate the structures of the obtained compounds (Supplementary Data). All pages of the Supporting Information file are numbered consecutively starting from title page with Figures S1–S30.

**Scheme 1:**
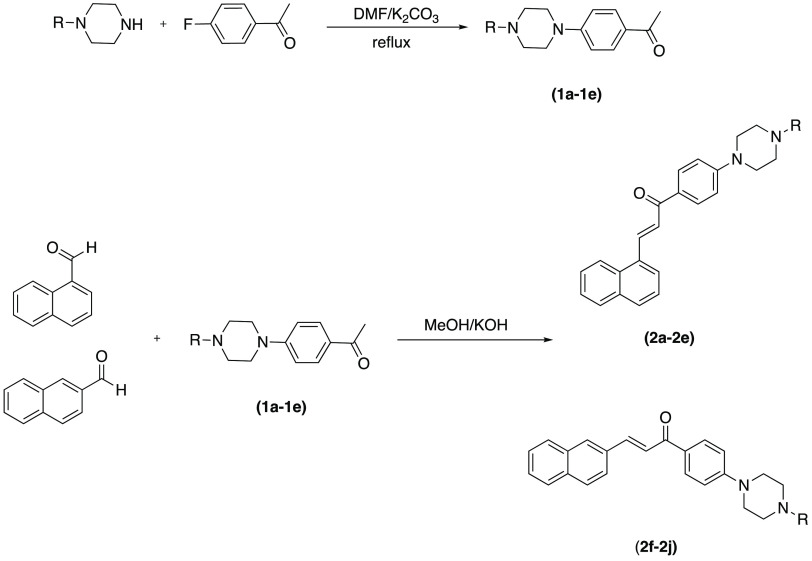
Synthesis Pathway for Obtained Compounds (**2a**–**2j**)

### 2.2 Cytotoxicity Test

The MTT test was performed using 3 different cell lines for calculated IC_50_ values of obtained compounds. The cytotoxicity results were presented in Table 1. None of the compounds showed activity against the HepG2 cell line. Among the compounds, only compound **2j** has an IC_50_ below 10 μM. Compound **2j** showed activity against A549 cell line with the value of IC_50_ = 7.8 ± 0.59 μM. In addition, compounds **2e** and **2i** showed activity against the A549 cell line with IC_50_ = 20.6 ± 0.52 μM and IC_50_ = 21.4 ± 2.6 μM, respectively. It is important that a compound does not show cytotoxic activity on a healthy cell, as well as showing activity on cancer cells. For this purpose, the cytotoxic effects of the compounds against the healthy mouse fibroblast cell (NIH3T3) were investigated. The most active compound, compound **2j**, showed cytotoxicity against the NIH3T3 cell line with an IC_50_ = 15.6 ± 0.8 μM. Calculating the selectivity index for this compound (IC_50_ value vs healthy cell/IC_50_ value vs cancer cell) is 1.990 against A549 cell line. Further testing was performed for compound **2j**, the most active compound in the series. The apoptotic potentials and anticancer activity mechanism of this compound were tried to be clarified.

**Table 1:** Antibacterial, Anticandidal, and Anticancer Activity of Synthesized Compounds (**2a**–**2j**) and Standard Drugs (**SD1–SD4**)

		Antibacterial activity						Anticandidal activity			Anticancer activity		
		MIC_50_ (μg/mL)^a^						MIC_50_ (μg/mL)^b^			IC_50_ (μM)^c^		
ID	R	*E*. *coli*	*P. aeruginosa*	*E. faecalis*	*B. subtilis*	*S. aureus*	*S. epidermis*	*C. albicans*	*C. krusei*	*C. parapsilopsis*	A549	HepG2	NIH3T3
**2a**	-Methyl	>100	62.5	31.3	>100	>100	62.5	31.3	62.5	62.5	>100	>100	61.9 ± 1.42
**2b**	-Ethyl	>100	62.5	31.3	>100	31.3	31.3	15.6	62.5	>100	>100	45.8 ± 8.40	22.9 ± 0.76
**2c**	-Isopropyl	>100	62.5	31.3	>100	62.5	31.3	15.6	62.5	>100	34.3 ± 5.56	96.1 ± 5.46	>100
**2d**	-Allyl	>100	62.5	15.7	>100	>100	31.3	31.3	62.5	>100	71.1 ± 3.08	45.1 ± 8.12	19.8 ± 1.31
**2e**	-2-Metoxyethyl	>100	62.5	31.3	>100	62.5	31.3	15.6	62.5	>100	20.6 ± 0.52	45.5 ± 2.50	19.8 ± 0.81
**2f**	-Methyl	>100	>100	31.3	>100	31.3	15.6	62.5	>100	>100	35.7 ± 0.37	29.7 ± 0.79	25.6 ± 0.26
**2g**	-Ethyl	>100	>100	31.3	>100	>100	62.5	31.3	>100	>100	39.1 ± 1.24	35.6 ± 0.98	52.4 ± 2.57
**2h**	-Isopropyl	>100	>100	31.3	>100	>100	62.5	31.3	>100	>100	41.3 ± 1.5	46.3 ± 1.25	66.5 ± 0.98
**2i**	-Allyl	>100	>100	31.3	>100	>100	31.3	31.3	>100	>100	21.4 ± 2.56	97.1 ± 4.94	>100
**2j**	–2-Metoxyethyl	>100	62.5	15.7	>100	31.3	31.3	15.6	15.6	>100	**7**.83 ± 0.**60**	38.9 ± 1.34	15.6 ± 0.75
**SD1**	-	<0.97	<0.97	<0.97	<0.97	<0.97	<0.97	-	-	-	-	-	-
**SD2**	-	-	-	-	-	-	-	3.90	3.90	1.95	-	-	-
**SD3**	-	-	-	-	-	-	-	7.81	7.81	3.90	-	-	-
**SD4**											2.97 ± 0.16	9.47 ± 1.49	>100

a The test results were expressed as means of triplicate assays.

b The test results were expressed as means of triplicate assays.

c The test results were expressed as means of quartet assays ± SEM. **SD1**: Azithromycin. **SD2**: Voriconazole. **SD3**: Fluconazole. **SD4**: Doxorubicin.

When the structures of the compounds are examined, it is seen that there are common naphthalene and piperazine rings in all compounds. The naphthalene ring is substituted from the first position (**2a**–**2e**) in some compounds and from the second position in some compounds. In the fourth position of the piperazine ring, compounds **2a** and **2f** contain methyl; compounds **2b** and **2g** contain ethyl; compounds **2c** and **2h** contain isopropyl; compounds **2d** and **2i** contain allyl; and compounds **2e** and **2j** contain 2-methoxyethyl substituent.

When the structure–activity relationships are examined, it is seen that the substitution of the naphthalene ring from the second position increases the activity. In addition, the idea that naphthalene ring contributes positively to the activity is strengthened by the aromatic hydrogen bonds it forms in the enzyme active sites. The incorporation of the 2-methoxyethyl substituent into the structure increases the activity. The two most active compounds in the series are the compounds that carry the 2-methoxyethyl substituent (**2e** and **2j**).

### 2.3 Antibacterial and Anticandidal Activity

Obtained compounds (**2a**–**2j**) were evaluated for antibacterial activity against *Escherichia coli* (*E*. *coli)* (ATCC 25922), *Pseudomonas aeruginosa* (*P*. *aeruginosa)* (ATCC 27853), *Escherichia faecalis* (*E*. *faecalis)* (ATCC 2942), *Bacillus subtilis* (*B*. *subtilis)* (ATCC 6051), *Staphylococcus aureus* (*S*. *aureus)* (ATCC 29213), and *Staphylococcus epidermidis (S*. *epidermidis)* (ATCC 12228). MIC_50_ values were determined via fluorometric measurements, using resazurin solution.^13^ Azithromycin was used as a standard drug in the antibacterial activity test. Results are presented in Table 1. When the antibacterial activity profile was examined, compound **2f** showed the highest activity against *S*. *epidermis* with a value of MIC_50_ = 15.6 μg/mL. Moreover, compounds **2d** and **2j** displayed the highest activity against *E*. *faecalis* with a value of MIC_50_ = 15.6 μg/mL.

Obtained compounds (**2a**–**2j**) were evaluated for anticandidal activity against *Candida albicans* (*C*. *albicans)* (ATCC 24433), *Candida krusei* (*C*. *krusei)* (ATCC 6258), and *Candida parapsilopsis* (*C*. *parapsilopsis)* (ATCC 22019*)*. MIC_50_ values were determined via fluorometric measurements, using resazurin solution.^13^ Voriconazole and fluconazole were used as a standard drug in the anticandidal activity test. Results are presented in Table 1. When the anticandidal activity profile was examined, compounds **2b**, **2c**, **2e**, and **2j** showed the highest activity against *C*. *albicans* with a value of MIC_50_ = 15.6 μg/mL. Additionally, compound **2j** displayed the highest activity against *C*. *krusei* with a value of MIC_50_ = 15.6 μg/mL. In general, it is seen that the 2-methoxyethyl substituent increases the anticandidal activity. When the substitution of naphthalene in its first position and its substitution from its second position is compared, the derivative containing 2-substituted naphthalene (compound **2j**) showed activity on two *Candida* lines.

### 2.4 Flow Cytometric Analysis

**Figure 2:**
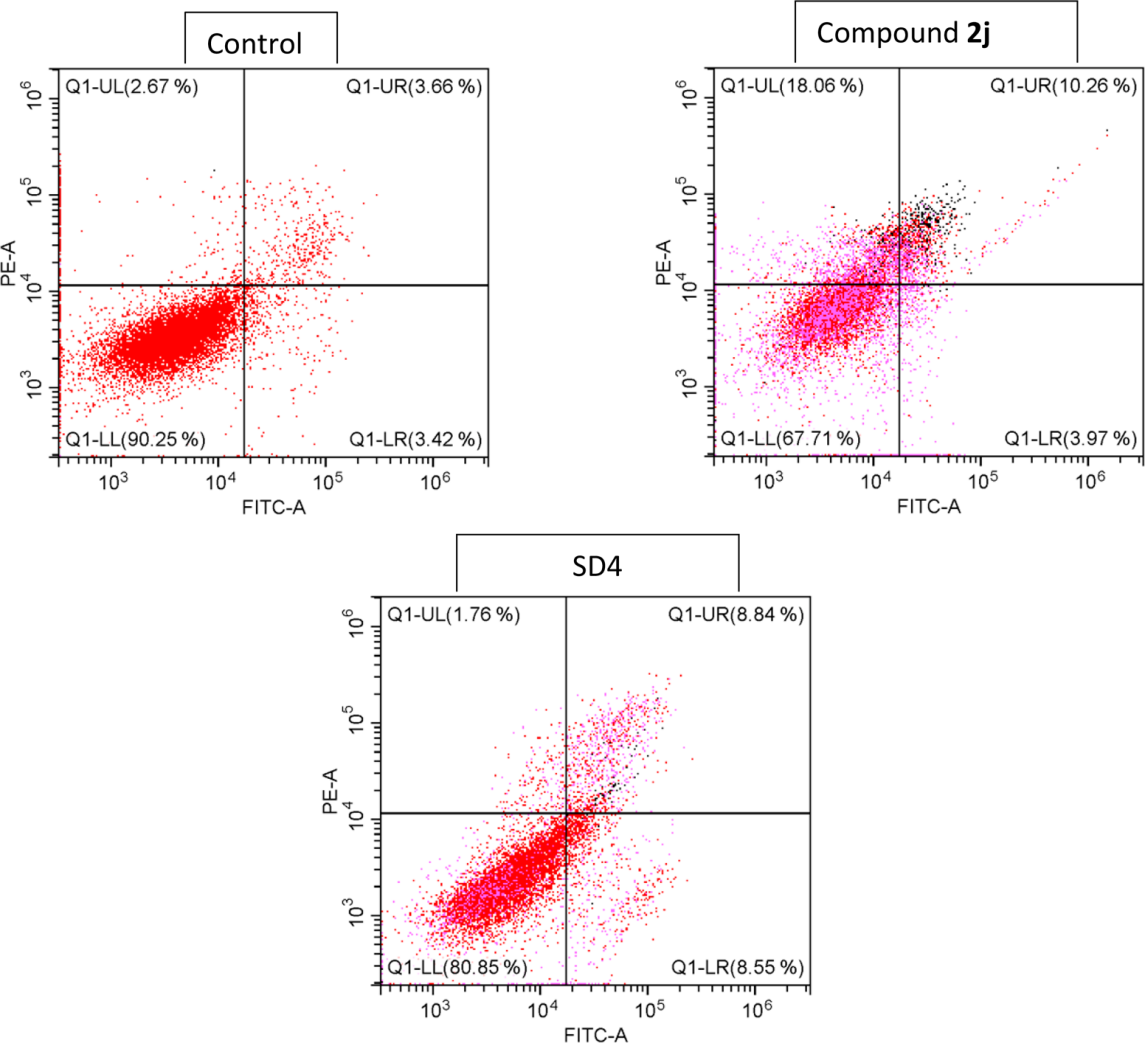
Flow cytometric analysis quadrants of compound **2j** and SD4.

Using a flow cytometer, gating was performed on a particular labeled cell, designated as Parent and abbreviated as “P” to demonstrate the activity. The flow cytometer was used to calculate the percentages of apoptotic and necrotic cells in four different quadrants, which are denoted by the letters “UL, UR, LL, LR” (Upper Left, Upper Right, Lower Left, Lower Right). Results of the flow cytometry analysis are shown in Figure 2. The four distinct quadrants in the diagram represent the following areas, respectively: Apoptotic or early apoptotic cells, LR: CF488A+/(EthD-III)-; necrotic or late apoptotic cells, UL: CF488A+/(EthD-III)+; intact cells, LL: CF488A/(EthD-III); and dead or necrotic cells, UR: CF488A+/(EthD-III)+. The IC_50_ concentration of each substance was used in administration.

Compound **2j** caused 18.1% necrosis, 10.3% late apoptosis, and 3.97% early apoptosis. All cells exposed to the chosen substance have been seen to activate the apoptotic process. Taking into account all flow cytometry findings, it was found that **2j** was the molecule that caused both cell types to undergo the maximum apoptosis. Compared to the SD4, they triggered apoptosis at a similar rate (Doxorubicin). The rate of apoptosis caused by compound **2j** was 14.2% (3.97% early apoptosis, 10.3% late apoptosis), while the rate induced by SD4 was 17.4% (8.55% early apoptosis, 8.84% late apoptosis). However, compound **2j** also increased the likelihood of necrosis by 18.1%.

### 2.5 Analysis of Mitochondrial Membrane Potential (MMP) by Flow Cytometry

**Figure 3:**
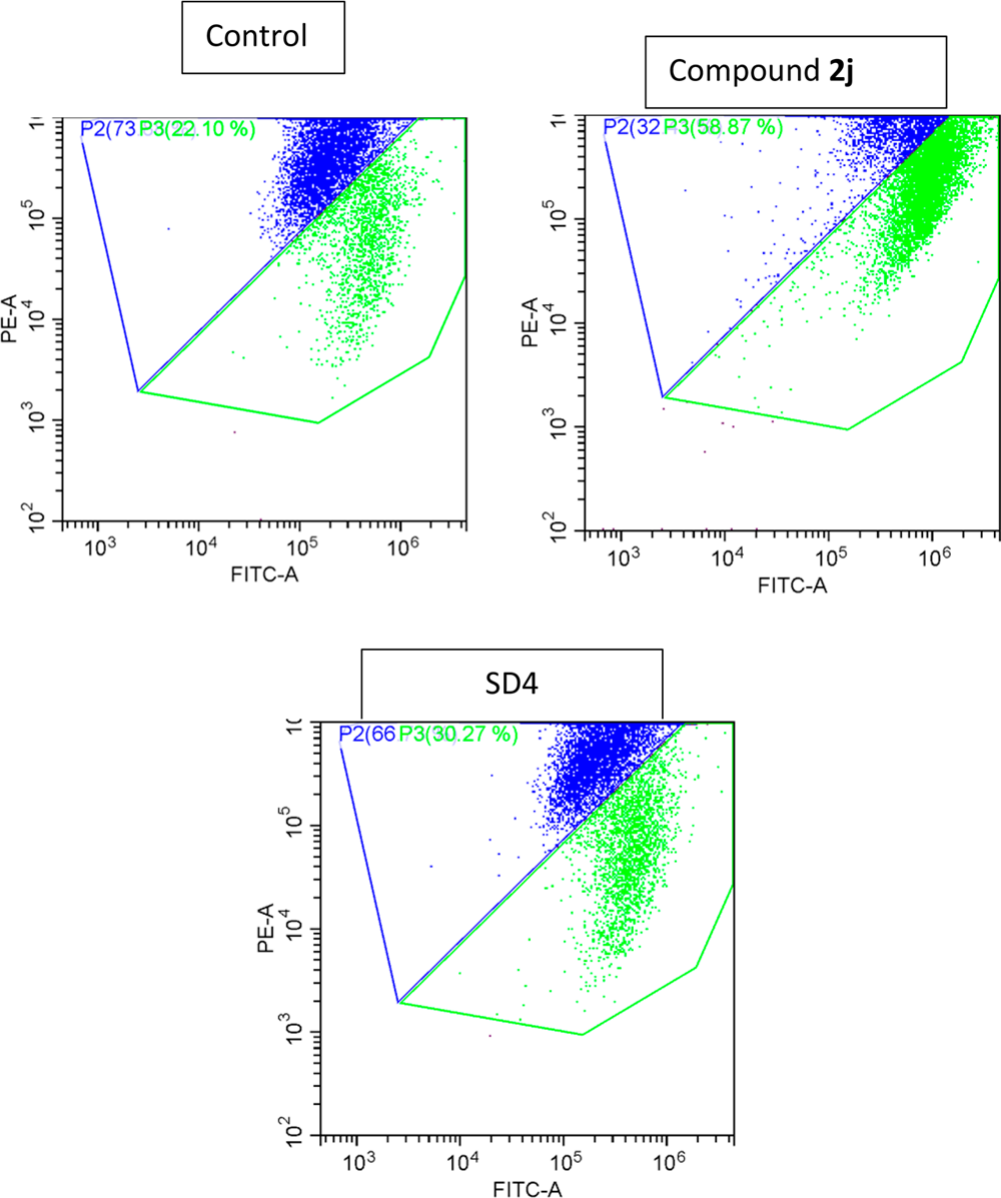
Analysis of mitochondrial membrane potential of compound **2j** and SD4.

Mitochondria-targeted agents play a very important role in the eradication of chemotherapy-resistant cancer cells. The most important reason for this is that mitochondria are key regulators of cell death. In addition, frequent changes in mitochondrial functions in neoplasia bring mitochondria-targeted drugs to the fore.^14^ For this purpose, the mitochondrial membrane potential of compound **2j** (the most active compound) was determined against A549 cell line by *in vitro* flow cytometric methods. Compound **2j** and doxorubicin were applied at IC_50_ concentration. After a 24-h incubation period, mitochondrial membrane potential was measured with JC-1 dye. The results obtained with the control group, compound **2j** and doxorubicin are presented in Figure 3. According to the results obtained, while doxorubicin showed 30.270% mitochondrial membrane potential; compound **2j** showed 58.870% mitochondrial membrane potential.

### 2.6 VEGFR-2 Inhibition Assay

The VEGFR-2 Kinase Assay Kit (Available from ref (15)) was used for the VEGFR-2 inhibition. The experiment was performed *in vitro* according to the kit procedure. Serial 11 dilutions of compound **2j** were prepared at concentrations of 1000 μM–0.01 μM. The IC_50_ value for compound **2j** was calculated using the kit procedure. According to the results obtained, the compound **2j** shows inhibitory activity on VEGFR-2 enzyme with the value of IC_50_ = 0.098 ± 0.005 μM.

### 2.7 *In Silico* Study

To justify potency improvement of the novel synthesized naphthalene-chalcone derivatives (**2a**–**2j**), molecular docking was conducted to investigate the potential binding mode of the most potent inhibitor (compound **2j**). Docking studies were performed on the VEGFR-2 crystals (PDB ID: 4ASE and PDB ID: 4ASD),^16^ as well as Caspase-3 (PDB ID: 4QTX),^17^ to demonstrate the caspase contribution of the apoptotic pathway.

**Figure 4:**
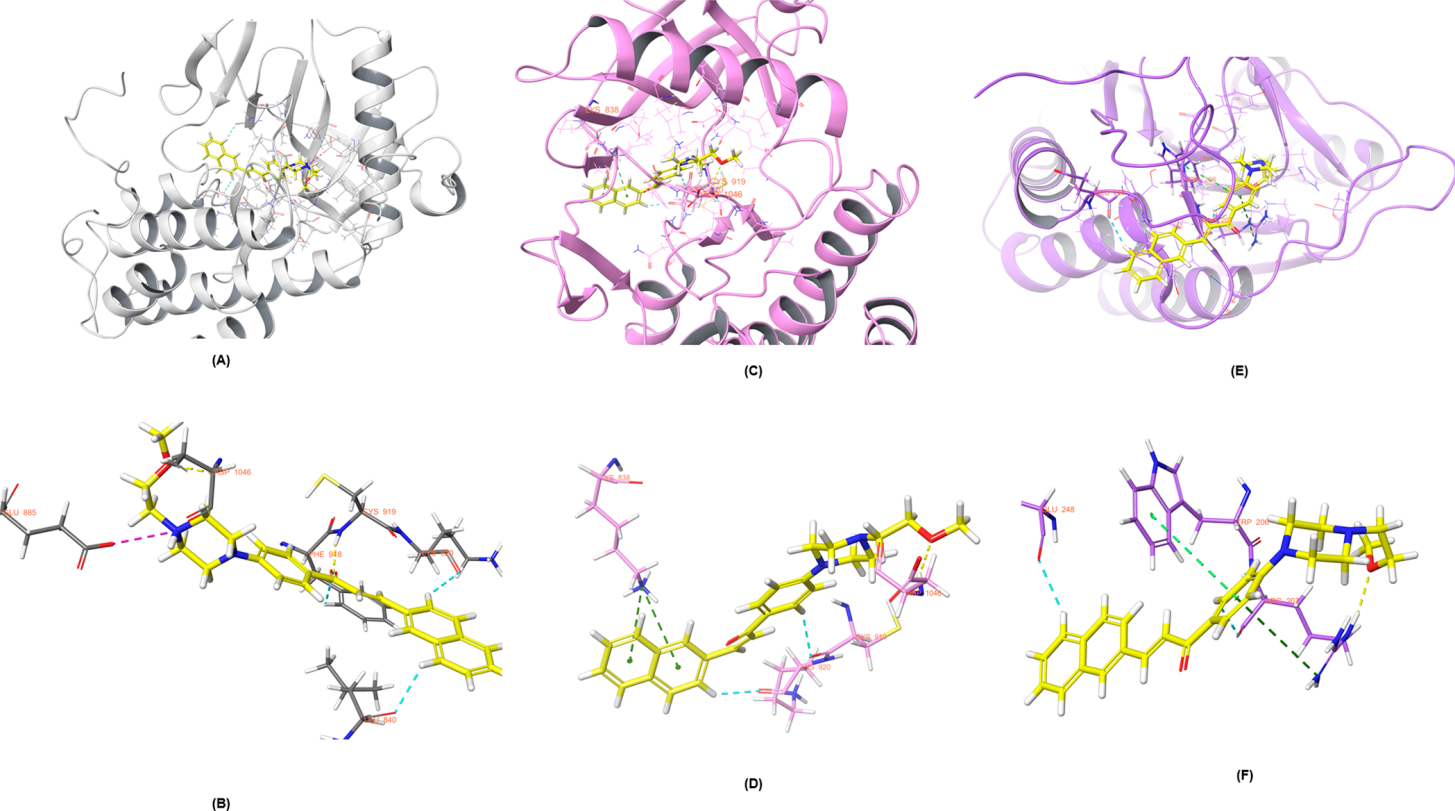
Molecular docking of VEGFR-2 enzymes (PDB code: 4ASE and PDB Code: 4ASD) and Caspase-3 enzyme (PDB code: 4QTX) with compound **2j**. (A) The three-dimensional interacting mode of compound **2j** in the active region of VEGFR-2 enzyme (PDB ID: 4ASE). (B) 3D docking pose of compound **2j** with the key amino acids within the binding pocket of 4ASE is shown: aromatic hydrogen bonds with blue dashed lines, hydrogen bonds with yellow dashed lines, and salt bridge with purple dashed lines. (C) The three-dimensional interacting mode of compound **2j** in the active region of VEGFR-2 enzyme (PDB ID: 4ASD). (D) 3D docking pose of compound **2j** with the key amino acids within the binding pocket of 4ASD is shown: aromatic hydrogen bonds with blue dashed lines, hydrogen bonds with yellow dashed lines, and salt bridge with purple dashed lines. (E) The three-dimensional interacting mode of compound **2j** in the active region of Caspase-3 enzyme (PDB ID: 4QTX). (F) 3D docking pose of compound **2j** with the key amino acids within the binding pocket of 4QTX is shown: aromatic hydrogen bonds with blue dashed lines, hydrogen bonds with yellow dashed lines and cation–pi interaction with dark green dashed lines, pi–pi interaction with green dashed lines.

Compound **2j**’s location in the VEGFR-2 enzyme’s active region is shown in Figure 4A. (PDB ID: 4ASE). The interaction with amino acids in the active site is shown in Figure 4B. Examining these interactions reveals a hydrogen connection between the amino group of Cys919 and the carbonyl group of molecule **2j**. The hydroxyl group of Asp1046 creates a salt bridge with the terminal nitrogen of the piperazine ring. The amine group of Glu885 and the unpaired electrons of the oxygen atom of the 2-methoxyethyl substituent form a hydrogen bond. In addition, the carbonyl groups of Leu840 and Lys920 were joined by aromatic hydrogen bonds between the hydrogens of the naphthalene ring.

The location of compound **2j** in the VEGFR-2 enzyme’s active region is depicted in Figure 4C. (PDB ID: 4ASD). The interaction with amino acids is depicted in Figure 4D for the active site. The naphthalene in compound **2j** establishes two pi-pi connections with the amino group of Lys838, according to the analysis of these interactions. The oxygen atom of the 2-methoxyethyl substituent’s unpaired electrons and the Asp1046 amine combine to create a hydrogen bond. Additionally, aromatic hydrogen bonds were created between the carbonyl groups of Lys920 and Cys919 by the hydrogens on their respective naphthalene and phenyl rings.

Compound **2j**’s location within the Caspase-3 enzyme’s active region is seen in Figure 4E. The interaction with amino acids in the active site is depicted in Figure 4F. When these interactions are analyzed, it becomes clear that the amino group of Arg207 and the oxygen atom of the 2-methoxyethyl substituent create a hydrogen bond. With the amino group of Arg207, the 1,4-disubstituted benzene interacts via a cation-pi interaction. With Trp206’s indole ring, the same ring interacts in a pi–pi fashion. Naphthalene ring hydrogens also created an aromatic hydrogen connection with Glu248’s carbonyl group.

## 3 Conclusion

The morbidity and mortality rates of cancer disease are increasing day by day. The side-effect profiles of the drugs used in the treatment and the resistance to the drugs create a need for new and effective drugs. Tyrosine kinase inhibitors have become a major part of cancer treatment in recent years. The advantages of the patient such as ease of use and target-oriented treatment have brought this group of drugs to the fore. For this purpose, within the scope of this study, new naphthalene-chalcone derivatives were synthesized and their anticancer effects were investigated. During cancer treatment, patients become vulnerable to bacterial and fungal infections. For this purpose, the fact that a drug with anticancer activity also shows antibacterial and anticandidal activity makes that drug more valuable.

Among the compounds obtained, compound **2j** (1-(4-(4-(2-methoxyethyl) piperazin-1-yl) phenyl)-3-(naphthalen-2-yl)prop-2-en-1-one) A549 cell showed activity with an IC_50_ = 7.835 ± 0.598 μM value against the line. This compound also has MIC_50_ = 15.625 μg/mL against *C*. *albicans* and *C*. *krusei*; it showed activity against *S*. *aureus* and *S. epidermis* with a value of MIC_50_ = 31.250 μg/mL.

## 4 Experimental Section

### 4.1 Chemistry

All the chemicals were obtained from industrial vendors and used without additional purification. Melting points (M.P.) were calculated using the uncorrected Mettler Toledo-MP90 Melting Point System. On a Bruker Fourier 300 (Bruker Bioscience, Billerica, MA, Germany), the ^1^H- and ^13^C NMR spectra were recorded in CDCl_3_ or DMSO-*d*_6_, respectively. On an LCMS-IT-TOF (Shimadzu, Kyoto, Japan) equipped with a PDA detector, mass spectra were recorded. The purity of the compounds was examined using Silica Gel 60 F254 with Thin-Layer Chromatography (Merck KGaA, Darmstadt, Germany).

#### 4.1.1 Synthesis of Keton Derivatives (**1a**–**1e**)

4-Fluoroacetophenone (0.88 mL, 72 mmol) was dissolved in DMSO (5 mL). Piperazine derivatives (72 mmol) was added in reaction mixture (potassium carbonate was used as catalysts). The reaction mixture was refluxed for 36 h. After TLC control, the reaction mixture was cooled and poured into iced water. Then, the obtained solution was extracted 3 times with ethyl acetate in the presence of sodium bicarbonate.

#### 4.1.2 General Procedures of Target Compounds (**2a**–**2j**)

The %40 KOH solution was prepared in MeOH (>99.5%). Appropriate obtained ketones (0.001 mol) were dissolved in the solution of KOH, and the obtained reaction mixture was stirred in room temperature for 30 min. Then the aldehydes (1-naftaldehyde or 2-naftaldehyde) were added in reaction mixture. After completion of the reaction, the precipitated product was filtered, washed with MeOH, and dried.

##### 4.1.2.1 1-(4-(4-Methylpiperazin-1-yl)phenyl)-3-(naphthalen-1-yl)prop-2-en-1-one (**2a**)

Yield: 89%, M.P.: 150.2–152.1 °C. ^1^H NMR (300 MHz, CDCl_3_): δ = 2.36 (3H, −CH_3_), 2.53–2.57 (4H, m, piperazine), 3.38–3.41 (4H, m, piperazine), 6.12 (1H, d, *J* = 9.6 Hz, Ar–H), 6.86 (2H, d, *J* = 9.1 Hz, Ar–H), 7.50–7.57 (3H, m, Ar–H), 7.64–7.69 (1H, m, Ar–H), 7.81–7.86 (3H, m, Ar–H), 7.89–7.93 (2H, m, Ar–H), 8.05–8.08 (1H, m, Ar–H). ^13^C NMR (75 MHz, CDCl_3_): δ = 45.9, 46.2, 47.1, 54.7, 103.1, 113.3, 122.9, 125.1, 125.5, 125.7, 127.9, 129.0, 130.4, 130.5, 145.1, 154.5, 155.9, 182.8. HRMS (*m*/*z*): [M + H]^+^ calcd for C_24_H_24_N_2_O: 357.1961; found: 357.1961.

##### 4.1.2.2 1-(4-(4-Ethylpiperazin-1-yl)phenyl)-3-(naphthalen-1-yl)prop-2-en-1-one (**2b**)

Yield: 87%, M.P.: 164.6–166.1 °C. ^1^H NMR (300 MHz, CDCl_3_): δ = 1.16 (3H, t, *J* = 7.2 Hz, −CH_3_), 2.50 (2H, q, *J* = 7.5 Hz, −CH_2_−), 2.62 (4H, t, *J* = 5.1 Hz, piperazine), 3.44 (4H, t, *J* = 5.1 Hz, piperazine), 6.96 (2H, d, *J* = 9.0 Hz, Ar–H), 7.51–7.61 (3H, m, Ar–H), 7.67 (1H, d, *J* = 15.3 Hz, Ar–H), 7.89–7.94 (3H, m, Ar–H), 8.06 (2H, d, *J* = 9.0 Hz, Ar–H), 8.30 (1H, d, *J* = 8.2 Hz, Ar–H), 8.65 (1H, m, *J* = 15.4 Hz, Ar–H). ^13^C NMR (75 MHz, CDCl_3_): δ = 12.0, 47.3, 52.4, 52.5, 113.5, 123.7, 124.8, 124.9, 125.5, 126.2, 126.8, 128.1, 128.7, 130.4, 130.8, 131.8, 132.9, 133.7, 140.1, 154.2, 187.9. HRMS (*m*/*z*): [M + H]^+^ calcd for C_25_H_26_N_2_O: 371.2118; found: 371.2129.

##### 4.1.2.3 1-(4-(4-Isopropylpiperazin-1-yl)phenyl)-3-(naphthalen-1-yl)prop-2-en-1-one (**2c**)

Yield: 81%, M.P.: 146.8–147.5 °C. ^1^H NMR (300 MHz, DMSO-*d*_6_): δ = 0.98 (6H, d, *J* = 6.5 Hz, −CH_3_), 2.50–2.55 (4H, m, piperazine), 2.61–2.68 (1H, m, −CH−), 3.32–3.35 (4H, m, piperazine), 7.00 (2H, d, *J* = 8.9 Hz, Ar–H), 7.59–7.64 (3H, m, Ar–H), 7.96–8.05 (3H, m, Ar–H), 8.07–8.10 (2H, m, Ar–H), 8.24 (2H, dd, *J*_1_ = 8.1 Hz, *J*_2_ = 14.3 Hz, Ar–H), 8.50 (1H, d, *J* = 15.3 Hz, Ar–H). ^13^C NMR (75 MHz, DMSO-*d*_6_): δ = 18.6, 47.3, 48.3, 54.1, 113.5, 123.5, 125.2, 125.9, 126.2, 126.7, 127.3, 127.6, 129.2, 130.9, 131.2, 131.6, 132.2, 133.8, 138.5, 154.5, 186.7. HRMS (*m*/*z*): [M + H]^+^ calcd for C_26_H_28_N_2_O: 385.2274; found: 385.2283.

##### 4.1.2.4 1-(4-(4-Allylpiperazin-1-yl) phenyl)-3-(naphthalen-1-yl)prop-2-en-1-one (**2d**)

Yield: 87%, M.P.: 120.2–121.9 °C. ^1^H NMR (300 MHz, CDCl_3_): δ = 2.51 (4H, t, *J* = 5.1 Hz, piperazine), 2.98 (2H, d, *J* = 6.6 Hz, Allyl-H), 3.32 (4H, t, *J* = 5.1 Hz, piperazine), 5.11–5.19 (2H, m, Allyl-H), 5.75–5.88 (1H, m, Allyl-H), 6.84 (2H, d, *J* = 9.0 Hz, Ar–H), 7.41–7.52 (3H, m, Ar–H), 7.57 (1H, d, *J* = 15.4 Hz, Ar–H), 7.79–7.84 (3H, m, Ar–H), 7.97 (2H, m, *J* = 8.9 Hz, Ar–H), 8.20 (1H, d, *J* = 8.1 Hz, Ar–H), 8.56 (1H, d, *J* = 15.4 Hz, Ar–H). ^13^C NMR (75 MHz, CDCl_3_): δ = 47.2, 51.7, 61.7, 113.5, 118.6, 123.7, 124.8, 124.9, 125.5, 126.2, 126.8, 128.1, 128.7, 130.4, 130.8, 131.8, 132.9, 133.7, 134.6, 140.1, 154.2, 187.9. HRMS (*m*/*z*): [M + H]^+^ calcd for C_26_H_26_N_2_O: 383.2118; found: 383.2126.

##### 4.1.2.5 1-(4-(4-(2-Methoxyethyl) piperazin-1-yl) phenyl)-3-(naphthalen-1-yl)prop-2-en-1-one (**2e**)

Yield: 85%, M.P.: 136.2–137.8 °C. ^1^H NMR (300 MHz, CDCl_3_): δ = 2.54–2.61 (6H, m, piperazine+CH_2_), 3.31 (3H, s, −OCH_3_), 3.37 (4H, t, *J* = 5.2 Hz, piperazine), 3.49 (2H, t, *J* = 5.4 Hz, −CH_2_−), 6.86 (2H, d, *J* = 9.1 Hz, Ar–H), 7.45–7.50 (3H, m, Ar–H), 7.58 (1H, d, *J* = 15.4 Hz, Ar–H), 7.80–7.85 (3H, m, Ar–H), 7.98 (2H, d, *J* = 9.0 Hz, Ar–H), 8.22 (1H, d, *J* = 8.2 Hz, Ar–H), 8.57 (1H, d, *J* = 15.4 Hz, Ar–H). ^13^C NMR (75 MHz, CDCl_3_): δ = 47.1, 53.2, 57.9, 59.0, 70.1, 113.5, 123.7, 124.8, 124.9, 125.5, 126.2, 126.8, 128.1, 128.7, 130.3, 130.8, 131.8, 132.9, 133.7, 140.1, 154.2, 187.9. HRMS (*m*/*z*): [M + H]^+^ calcd for C_26_H_28_N_2_O_2_: 401.2224; found: 401.2238.

##### 4.1.2.6 1-(4-(4-Methylpiperazin-1-yl) phenyl)-3-(naphthalen-2-yl)prop-2-en-1-one (**2f**)

Yield: 90%, M.P.: 204.5–206.2 °C. ^1^H NMR (300 MHz, CDCl_3_): δ = 2.38 (3H, s, −CH_3_), 2.59 (4H, t, *J* = 5.1 Hz, piperazine), 3.43 (4H, t, *J* = 5.1 Hz, piperazine), 6.96 (2H, d, *J* = 9.0 Hz, Ar–H), 7.52–7.55 (2H, m, Ar–H), 7.70 (1H, d, *J* = 15.6 Hz, Ar–H), 7.84–7.90 (4H, m, Ar–H), 7.98 (1H, d, *J* = 15.6 Hz, Ar–H), 7.95–8.00 (2H, m, Ar–H). ^13^C NMR (75 MHz, CDCl_3_): δ = 46.2, 47.3, 54.8, 113.3, 113.6, 122.1, 123.8, 126.7, 127.2, 127.8, 128.3, 128.6, 130.3, 130.7, 132.8, 133.4, 134.2, 143.3, 154.1, 188.0. HRMS (*m*/*z*): [M + H]^+^ calcd for C_24_H_24_N_2_O: 357.1961; found: 357.1971.

##### 4.1.2.7 1-(4-(4-Ethylpiperazin-1-yl) phenyl)-3-(naphthalen-2-yl)prop-2-en-1-one (**2g**)

Yield: 90%, M.P.: 196.5–198.0 °C. ^1^H NMR (300 MHz, CDCl_3_): δ = 1.08 (3H, t, *J* = 7.2 Hz, CH_3_), 2.42 (2H, q, *J* = 7.1 Hz, −CH_2_−), 2.53–2.56 (4H, m, piperazine), 6.88 (2H, d, *J* = 9.1 Hz, Ar–H), 7.43–7.47 (2H, m, Ar–H), 7.62 (1H, d, *J* = 15.6 Hz, Ar–H), 7.75–7.81 (4H, m, Ar–H), 7.89 (1H, d, *J* = 15.6 Hz, Ar–H), 7.97 (3H, d, *J* = 9.0 Hz, Ar–H). ^13^C NMR (75 MHz, CDCl_3_): δ = 12.0, 47.3, 52.4, 52.5, 113.5, 113.5, 122.1, 123.8, 126.7, 127.1, 127.8, 128.2, 128.6, 130.3, 130.7, 132.8, 133.4, 134.2, 143.2, 154.1, 188.0. HRMS (*m*/*z*): [M + H]^+^ calcd for C_25_H_26_N_2_O: 371.2118; found: 371.2119.

##### 4.1.2.8 1-(4-(4-Isopropylpiperazin-1-yl) phenyl)-3-(naphthalen-2-yl)prop-2-en-1-one (**2h**)

Yield: 88%, M.P.: 213.3–214.9 °C. ^1^H NMR (300 MHz, CDCl_3_): δ = 1.03 (6H, d, *J* = 6.5 Hz, −CH_3_), 2.61 (4H, t, *J* = 5.0 Hz, piperazine), 2.65–2.71 (1H, m, −CH−), 3.34 (4H, t, *J* = 5.0 Hz, piperazine), 6.87 (2H, d, *J* = 8.9 Hz, Ar–H), 7.43–7.46 (2H, m, Ar–H), 7.62 (1H, d, *J* = 15.6 Hz, Ar–H), 7.72–7.83 (4H, m, Ar–H), 7.89 (1H, d, *J* = 15.6 Hz, Ar–H), 7.97 (3H, d, *J* = 8.9 Hz, Ar–H). ^13^C NMR (75 MHz, CDCl_3_): δ = 18.6, 47.6, 48.5, 54.5, 113.4, 113.4, 122.2, 123.8, 126.7, 127.1, 127.8, 128.1, 128.6, 130.3, 130.7, 132.8, 133.4, 134.2. HRMS (*m*/*z*): [M + H]^+^ calcd for C_26_H_28_N_2_O: 385.2274; found: 385.2285.

##### 4.1.2.9 1-(4-(4-Allylpiperazin-1-yl) phenyl)-3-(naphthalen-2-yl)prop-2-en-1-one (**2i**)

Yield: 83%, M.P.: 186.9–188.8 °C.^1^H NMR (300 MHz, DMSO-*d*_6_): δ = 2.54 (4H, t, *J* = 5.0 Hz, piperazine), 3.00 (2H, d, *J* = 6.5 Hz, Allyl-H), 3.34 (4H, t, *J* = 5.1 Hz, piperazine), 5.12–5.21 (2H, m, Allyl-H), 5.76–5.88 (1H, m, Allyl-H), 6.87 (2H, d, *J* = 9.0 Hz, Ar–H), 7.43–7.47 (2H, m, Ar–H), 7.62 (1H, d, *J* = 15.6 Hz, Ar–H), 7.75–7.81 (4H, m, Ar–H), 7.89 (1H, d, *J* = 15.6 Hz, Ar–H), 7.97 (3H, d, *J* = 8.9 Hz, Ar–H). ^13^C NMR (75 MHz, DMSO-*d*_6_): δ = 47.3, 52.7, 61.8, 113.5, 113.5, 118.6, 122.1, 123.8, 126.7, 127.2, 127.8, 128.3, 128.6, 130.3, 130.7, 132.8, 133.4, 134.2, 134.6, 143.2, 154.1, 188.0. HRMS (*m*/*z*): [M + H]^+^ calcd for C_26_H_26_N_2_O: 383.2118; found: 383.2124.

##### 4.1.2.10 1-(4-(4-(2-Methoxyethyl) piperazin-1-yl)phenyl)-3-(naphthalen-2-yl)prop-2-en-1-one (**2j**)

Yield: 83%, M.P.: 164.4–166.3 °C. ^1^H NMR (300 MHz, CDCl_3_): δ = 2.55–2.62 (6H, m, piperazine+-CH_2_−), 3.32 (3H, s, −OCH_3_), 3.36 (4H, t, *J* = 5.1 Hz, piperazine), 3.50 (2H, t, *J* = 5.4 Hz, −CH_2_−), 6.87 (2H, d, *J* = 9.0 Hz, Ar–H), 7.44–7.47 (2H, m, Ar–H), 7.62 (1H, d, *J* = 15.6 Hz, Ar–H), 7.75–7.82 (4H, m, Ar–H), 7.89 (1H, d, *J* = 15.6 Hz, Ar–H), 7.97 (3H, d, *J* = 8.9 Hz, Ar–H). ^13^C NMR (75 MHz, CDCl_3_): δ = 47.1, 53.2, 53.3, 57.9, 70.1, 113.5, 113.5, 122.1, 123.8, 126.7, 127.2, 127.8, 128.2, 128.6, 128.7, 130.2, 130.7, 132.8, 133.4, 143.2, 154.2, 179.7. HRMS (*m*/*z*): [M + H]^+^ calcd for C_26_H_28_N_2_O_2_: 401.2224; found: 401.2237.

### 4.2 Cytotoxicity Test

The 3-(4,5-dimethylthiazol-2-yl)-2,5-diphenyltetrazolium salt reduction method, or MTT assay, is used to measure the metabolic activity of live cells. The formazan salt, which turns purple at the conclusion of the incubation period, allows for the spectrometric determination of the cell viability rate.^18^Table 1 shows findings of cell growth inhibition following a 24-h treatment with the resulting compounds for 4 different cell lines. MTT assays were used to screen compounds **2a**–**2j** for cytotoxicity. Doxorubicin is used as a therapeutic drug. MTT assays were carried out as previously explained.^19^

### 4.3 Antibacterial and Anticandidal Activity

The antimicrobial activity of obtained derivatives (**2a**–**2j**) was screened on six bacterial and three fungal strains according to the standard procedure of CLSI^20^ as described in the previous study.^21^ The antibacterial activities of the obtained compounds were screened against *B*. *subtilis* (ATCC 6051), *E*. *coli* (ATCC 25922), *E*. *faecalis* (ATCC 2942), *P*. *aeruginosa* (ATCC 27853), *S*. *aureus* (ATCC 29213), and *S*. *epidermidis* (ATCC 12228). The obtained compounds were evaluated for anticandidal activity against *C*. *albicans* (ATCC 24433), *C*. *krusei* (ATCC 6258), and *C*. *parapsilopsis* (ATCC 22019). For antibacterial activity, azithromycin was utilized as a reference drug, whereas voriconazole and fluconazole were used for anticandidal activity.

#### 4.3.1 Flow Cytometric Analysis

Death pathway of the carcinogenic cell lines was detected by Apoptosis, Necrosis and Healthy Cell Quantitation Kit Plus (Cat: 30066, Biotium, Hayward, CA, USA)^22^ as reported in the manufacturer’s instruction. The IC_50_ values for doxorubicin and compound **2j** were applied. Cells were collected by centrifugation at 1200 rpm for 5 min following a 24-h incubation period. It was washed 2 times with PBS, centrifuged and turned into pellets. One ×10^6^ cells/mL of annexin were suspended in V-FITC binding buffer. Ethidium iodide (5 mL), Hoestch 1 mL, and annexin V-FITC (5 mL) were added to stain cells and using the CytoFLEX Flow Cytometer (Beckman Coulter Life Sciences, USA) and CytExpert for CytoFLEX Acquisition and Analysis Software Version 2.2.0.97 instrument. Fluorescence measurements were made using a flow cytometer in accordance with the instrument procedure.

### 4.4 Analysis of Mitochondrial Membrane Potential (MMP) by Flow Cytometry

The BD Mitoscreen Mitochondrial Membrane Potential Detection JC-1 Kit (available from ref (23)) was used for the MMP test. First, A549 cells were seeded in 25 mL flasks and incubated for 24 h in a 5% CO_2_ incubator. At the end of the period, compound **2j** and doxorubicin were added to the flasks at IC_50_ concentrations, and the 24-h incubation period was started. At the end of this period, cells were collected and centrifuged in accordance with the kit contents. After the upper part was removed, JC-1 dye was added and incubated at 37 °C for 10–15 min. At the end of the period, it was washed 2 times with washing solution and reading was done with the appropriate procedure using the CytoFLEX Flow Cytometer (Beckman Coulter Life Sciences, USA) and CytExpert for CytoFLEX Acquisition and Analysis Software Version 2.2.0.97.

### 4.5 VEGFR-2 Inhibition Assay

The VEGFR-2 Kinase Assay Kit (available from ref (15)) was used for the VEGFR-2 inhibition. The experiment was performed *in vitro* according to the kit procedure.

### 4.6 *In Silico* Study

Molecular docking studies were performed using in-silico procedure to define the binding modes of compound **2j** (active compound) in the active regions of enzymes X-ray crystal structures of VEGFR-2 (PDB ID: 4ASE and PDB ID: 4ASD)^16^ and Caspase-3 (PDB ID: 4QTX)^17^ were retrieved from Protein Data Bank server (www.pdb.org, accessed 19 Sep 2022). The Schrödinger Maestro interface,^24^ Ligprep module,^25^ and Glide module^26^ were used for molecular docking procedures, and docking runs were performed in standard precision docking mode (SP).

## Supporting Information Available

^1^H NMR spectra of the compound **2a**; ^13^C NMR spectra of the compound **2a**; HRMS spectra of the compound **2a**; ^1^H NMR spectra of the compound **2b**; ^13^C NMR spectra of the compound **2b**; HRMS spectra of the compound **2b**; ^1^H NMR spectra of the compound **2c**; ^13^C NMR spectra of the compound **2c**; HRMS spectra of the compound **2c**; ^1^H NMR spectra of the compound **2d**; ^13^C NMR spectra of the compound **2d**; HRMS spectra of the compound **2d**; ^1^H NMR spectra of the compound **2e**; ^13^C NMR spectra of the compound **2e**; HRMS spectra of the compound **2e**; ^1^H NMR spectra of the compound **2f**; ^13^C NMR spectra of the compound **2f**; HRMS spectra of the compound **2f**; ^1^H NMR spectra of the compound **2g**; ^13^C NMR spectra of the compound **2g**; HRMS spectra of the compound **2g**; ^1^H NMR spectra of the compound **2h**; ^13^C NMR spectra of the compound **2h**; HRMS spectra of the compound **2h**; ^1^H NMR spectra of the compound **2i**; ^13^C NMR spectra of the compound **2i**; HRMS spectra of the compound **2i**; ^1^H NMR spectra of the compound **2j**; ^13^C NMR spectra of the compound **2j**; and HRMS spectra of the compound **2j** (PDF)

## References

[ref1] El Ghalia H.; Amina G.; El Aissouq A.; Oussama C.; Hicham E. H.; Abdelkrim O.; Mohammed B. A quantitative study of the structure-activity relationship and molecular docking of 5.6. 7-trimethoxy-N-aryl-2-styrylquinolin-4-amines as potential anticancer agents using quantum chemical descriptors and statistical methods. J. Mol. Struct. 2022, 1270, 133794 10.1016/j.molstruc.2022.133794.

[ref2] Qi B.; Wang F.; He H.; Fan M.; Hu L.; Xiong L.; Gong G.; Shi S.; Song X. Identification of (S)-1-(2-(2, 4-difluorophenyl)-4-oxothiazolidin-3-yl)-3-(4-((7-(3-(4-ethylpiperazin-1-yl) propoxy)-6-methoxyquinolin-4-yl) oxy)-3, 5-difluorophenyl) urea as a potential anti-colorectal cancer agent. Eur. J. Med. Chem. 2022, 239, 114561 10.1016/j.ejmech.2022.114561. 35763868

[ref3] Engle K.; Kumar G. Cancer multidrug-resistance reversal by ABCB1 inhibition: A recent update. Eur. J. Med. Chem. 2022, 239, 114542 10.1016/j.ejmech.2022.114542. 35751979

[ref4] JawalePatil P. D.; Bhamidipati K.; Damale M. G.; Sangshetti J. N.; Puvvada N.; Bhosale R. S.; Ingle R. D.; Pawar R. P.; Bhosale S. V.; Bhosale S. V. Synthesis of naphthalimide derivatives bearing benzothiazole and thiazole moieties: In vitro anticancer and in silico ADMET study. J. Mol. Struct. 2022, 1263, 133173 10.1016/j.molstruc.2022.133173.

[ref5] Pérez-Soto M.; Peñalver P.; Street S. T.; Weenink D.; O’Hagan M. P.; Ramos-Soriano J.; Jiang Y. J.; Hollingworth G. J.; Galan M. C.; Morales J. C. Structure-activity relationship studies on divalent naphthalene diimide G quadruplex ligands with anticancer and antiparasitic activity. Bioorg. Med. Chem. 2022, 71, 116946 10.1016/j.bmc.2022.116946. 35939903

[ref6] Waheed S. A.; Mustafa Y. F. Novel naphthalene-derived coumarin composites: synthesis, antibacterial, and antifungal activity assessments. Eurasian Chemical Communications 2022, 4 (8), 709–724. 10.22034/ecc.2022.335455.1396.

[ref7] Tuğrak M.; Yamali C.; Gül H. İ.; Demir Y. Inhibitory effects of the chalcones towards carbonic anhydrase I, II and acetylcholinesterase enzymes. Erzincan Üniversitesi Fen Bilimleri Enstitüsü Dergisi 2020, 13 (3), 1138–1146. 10.18185/erzifbed.748798.

[ref8] Tukur A.; Habila J. D.; Ayo R. G.-O.; Iyun O. R. A. Synthesis, characterization and antibiotic evaluation of some novel (E)-3-(4-diphenylamino) phenyl)-1-(4′-fluorophenyl) prop-2-en-1-one chalcones and their analogues. Beni-Suef Univ. J. Basic Appl. Sci. 2022, 11 (1), 66 10.1186/s43088-022-00246-8.

[ref9] Qin T.; Ma Y.-Y.; Dong C.-E.; Wu W.-L.; Feng Y.-Y.; Yang S.; Su J.-B.; Si X.-X.; Wang X.-J.; Shi D.-H. Design, synthesis, cytotoxicity evaluation and molecular docking studies of 1, 4-naphthoquinone derivatives. J. Mol. Struct. 2022, 1263, 133067 10.1016/j.molstruc.2022.133067.

[ref10] Yousef R. G.; Eldehna W. M.; Elwan A.; Abdelaziz A. S.; Mehany A.; Gobaara I. M.; Alsfouk B. A.; Elkaeed E. B.; Metwaly A. M.; Eissa I. Design, Synthesis, In Silico and In Vitro Studies of New Immunomodulatory Anticancer Nicotinamide Derivatives Targeting VEGFR-2. Molecules 2022, 27 (13), 4079 10.3390/molecules27134079. 35807326 PMC9268560

[ref11] Alsaif N. A.; Mahdy H. A.; Alanazi M. M.; Obaidullah A. J.; Alkahtani H. M.; Al-Hossaini A. M.; Al-Mehizi A. A.; Elwan A.; Taghour M. S. Targeting VEGFR-2 by new quinoxaline derivatives: Design, synthesis, antiproliferative assay, apoptosis induction, and in silico studies. Arch. Pharm. 2022, 355 (2), 2100359 10.1002/ardp.202100359. 34862634

[ref12] Yousef R. G.; Elwan A.; Gobaara I. M.; Mehany A. B.; Eldehna W. M.; El-Metwally S. A.; A. Alsfouk B.; Elkaeed E. B.; Metwaly A. M.; Eissa I. H. Anti-cancer and immunomodulatory evaluation of new nicotinamide derivatives as potential VEGFR-2 inhibitors and apoptosis inducers: in vitro and in silico studies. J. Enzyme Inhibit. Med. Chem. 2022, 37 (1), 2206–2222. 10.1080/14756366.2022.2110868. PMC9466619 35980113

[ref13] Borra R. C.; Lotufo M. A.; Gagioti S. M.; Barros F. d. M.; Andrade P. M. A simple method to measure cell viability in proliferation and cytotoxicity assays. Braz. Oral. Res. 2009, 23, 255–262. 10.1590/S1806-83242009000300006. 19893959

[ref14] Fulda S.; Galluzzi L.; Kroemer G. Evasion of cell death is a hallmark of human cancers and a major cause of treatment failure. Nat. Rev. Drug Discov 2010, 9, 447–464. 10.1038/nrd3137. 20467424

[ref15] https://bpsbioscience.com/vegfr2-kdr-kinase-assay-kit-40325 (accessed by 01-09-2022).

[ref16] McTigue M.; Murray B. W.; Chen J. H.; Deng Y.-L.; Solowiej J.; Kania R. S. Molecular conformations, interactions, and properties associated with drug efficiency and clinical performance among VEGFR TK inhibitors. PNAS. 2012, 109 (45), 18281–18289. 10.1073/pnas.1207759109. 22988103 PMC3494931

[ref17] Cade C.; Swartz P.; MacKenzie S. H.; Clark A. C. Modifying caspase-3 activity by altering allosteric networks. Biochemistry 2014, 53 (48), 7582–7595. 10.1021/bi500874k. 25343534 PMC4263430

[ref18] Berridge M. V.; Herst P. M.; Tan A. S. Tetrazolium dyes as tools in cell biology: new insights into their cellular reduction. Biotechnol. Annu. Rev. 2005, 11, 127–152. 10.1016/S1387-2656(05)11004-7. 16216776

[ref19] Osmaniye D.; Levent S.; Ardıç C. M.; Atlı Ö.; Özkay Y.; Kaplancıklı Z. A. Synthesis and anticancer activity of some novel benzothiazole-thiazolidine derivatives. Phosphorus Sulfur Silicon Relat. Elem. 2018, 193 (4), 249–256. 10.1080/10426507.2017.1395878.

[ref20] Wikler M. A. Methods for dilution antimicrobial susceptibility tests for bacteria that grow aerobically: approved standard. Clsi (Nccls) 2006, 26, M7–A7.

[ref21] Evren A. E.; Dawbaa S.; Nuha D.; Yavuz Ş. A.; Gül Ü. D.; Yurttaş L. Design and synthesis of new 4-methylthiazole derivatives: In vitro and in silico studies of antimicrobial activity. J. Mol. Struct. 2021, 1241, 130692 10.1016/j.molstruc.2021.130692.

[ref22] https://bpsbioscience.com/vegfr2-kdr-kinase-assay-kit-40325 (accessed by 01-08-2022).

[ref23] https://www.bdbiosciences.com/en-nz/products/reagents/flow-cytometry-reagents/research-reagents/panels-multicolor-cocktails-ruo/mitoscreen-jc-1.551302 (accessed by 01-08-2022).

[ref24] Schrödinger. Maestro, release 2020-3; Schrödinger, LLC: New York, NY, USA, 2020.

[ref25] Schrödinger. LigPrep 2020, release 2020-1; Schrödinger, LLC: New York, NY, USA, 2020.

[ref26] Schrödinger. Glide, release 2020-3; Schrödinger, LLC: New York, NY, USA, 2020.

