# Supplementary material for: Design, Synthesis, and Biological Evaluation Studies of Novel Naphthalene-Chalcone Hybrids As Antimicrobial, Anticandidal, Anticancer, and VEGFR-2 Inhibitors
Source: ACS Omega. 2023 Feb 13;8(7):6669–78. doi: 10.1021/acsomega.2c07256 (PMC9947975; doi:10.1021/acsomega.2c07256)
Supplement: Supplementary file 1 — ao2c07256_si_001.pdf [file ao2c07256_si_001.pdf]

Design, synthesis, and biological evaluation studies of novel naphthalene-chalcone hybrids as antimicrobial, anticandidal, anticancer and VEGFR-2 inhibitors

Derya Osmaniye[1,2*], Begüm Nurpelin Sağlık[1,2], Narmin Khalilova[1], Serkan Levent[1,2], Gizem Bayazıt[3], Ülküye Dudu Gül[3], Yusuf Özkay[1,2], Zafer Asım Kaplancıklı[1]

1 Department of Pharmaceutical Chemistry, Faculty of Pharmacy, Anadolu University, 26470 Eskişehir, Turkey
2 Central Analysis Laboratory, Faculty of Pharmacy, Anadolu University, 26470 Eskişehir, Turkey
3 Vocational School of Health Services, Biotechnology Application and Research Center, Bilecik Seyh Edebali University, Bilecik, Turkey

*Correspondences:
Dr. Derya Osmaniye, Department of Pharmaceutical Chemistry, Faculty of Pharmacy, Anadolu University, 26470 Eskişehir, Turkey
Email: dosmaniye@anadolu.edu.tr

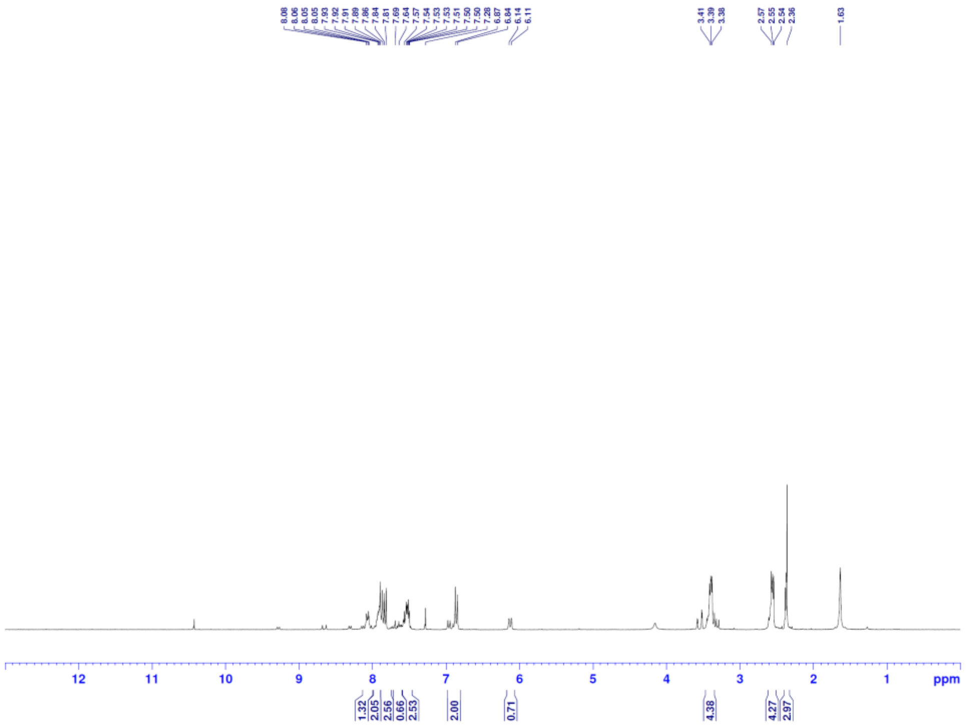

Figure S1. ^{1}H-NMR spectra of the compound **2a**

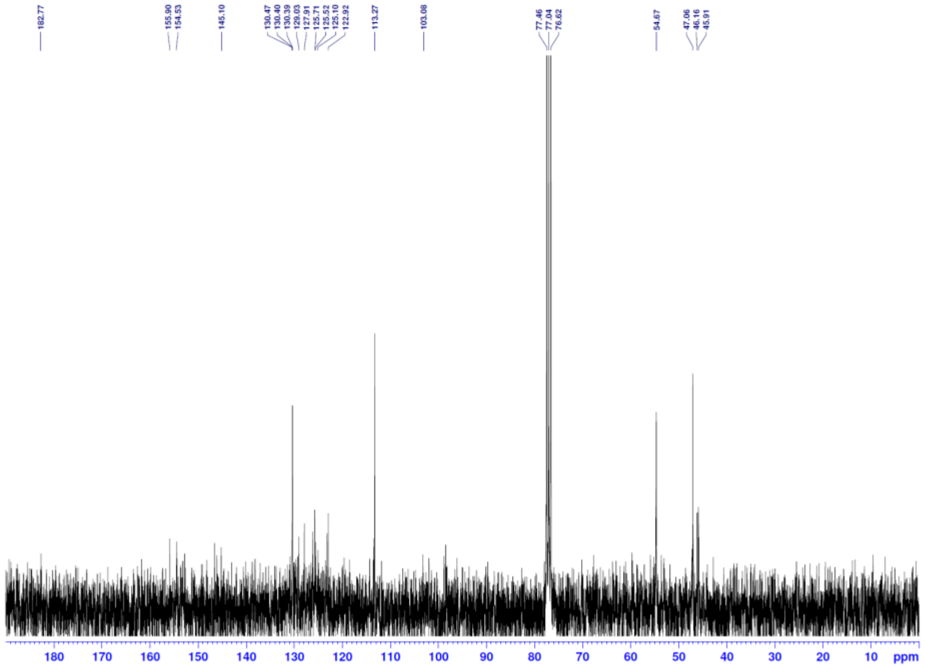

Figure S2. ^{13}C-NMR spectra of the compound **2a**

Formula Predictor Report - NK1-1_127.lcd

Page 1 of 1

Data File: C:\LabSolutions\Data\Analiz\derya\NK1-1_127.lcd

Elmt	Val.	Min	Max	Elmt	Val.	Min	Max	Elmt	Val.	Min	Max	Elmt	Val.	Min	Max	Use Adduct
H	1	9	40	O	2	1	4	S	2	0	0	Ru	2	0	0	H
C	4	10	40	F	1	0	0	Cl	1	0	0	Pd	2	0	0	
N	3	1	5	P	3	0	0	Br	1	0	0	I	3	0	0	

Error Margin (ppm):	5	DBE Range:	5.0 - 25.0	Electron Ions:	both
HC Ratio:	unlimited	Apply N Rule:	yes	Use MSn Info:	yes
Max Isotopes:	3	Isotope RI (%):	1.00	Isotope Res:	9000
MSn Iso RI (%):	10.00	MSn Logic Mode:	AND	Max Results:	150

Event#: 1 MS(E+) Ret. Time : 2.613 Scan# : 393

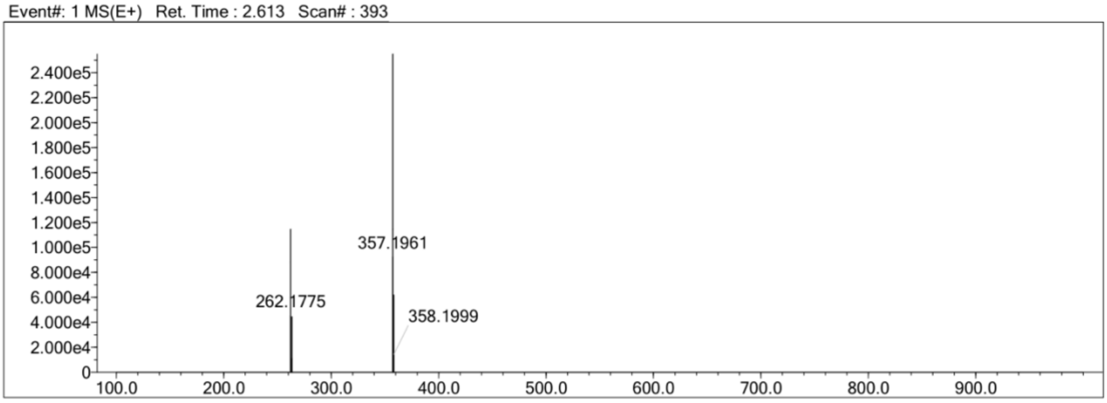

Measured region for 357.1961 m/z

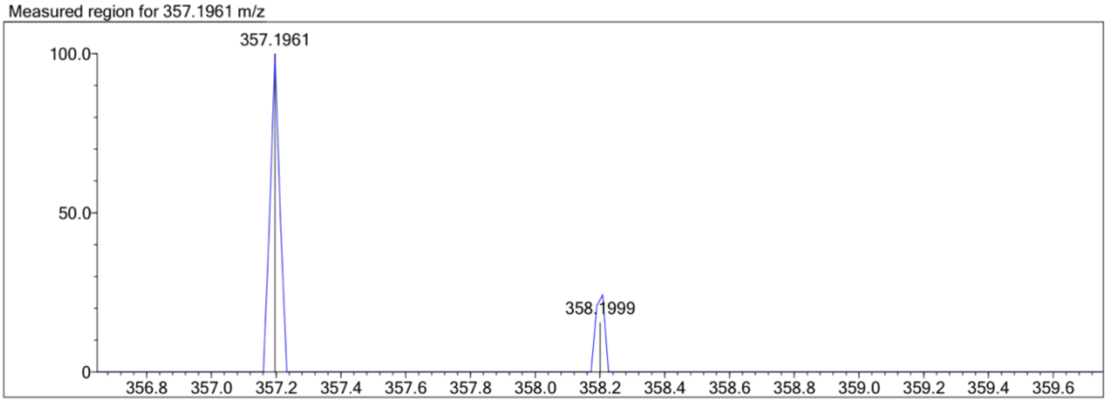

C24 H24 N2 O [M+H]+ : Predicted region for 357.1961 m/z

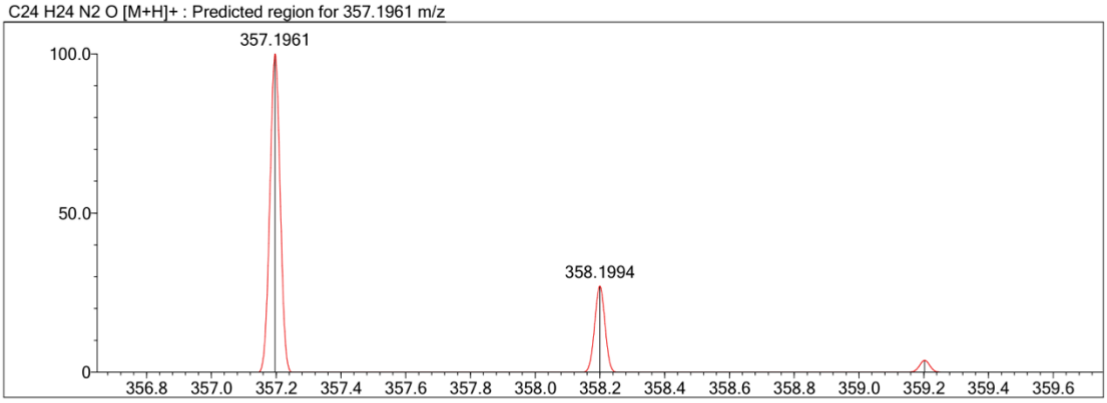

Rank	Score	Formula (M)	Ion	Meas. m/z	Pred. m/z	Df. (mDa)	Df. (ppm)	Iso	DBE
1	85.34	C24 H24 N2 O	[M+H]+	357.1961	357.1961	-0.0	0.00	85.34	14.0

Figure S3. HRMS spectra of the compound **2a**

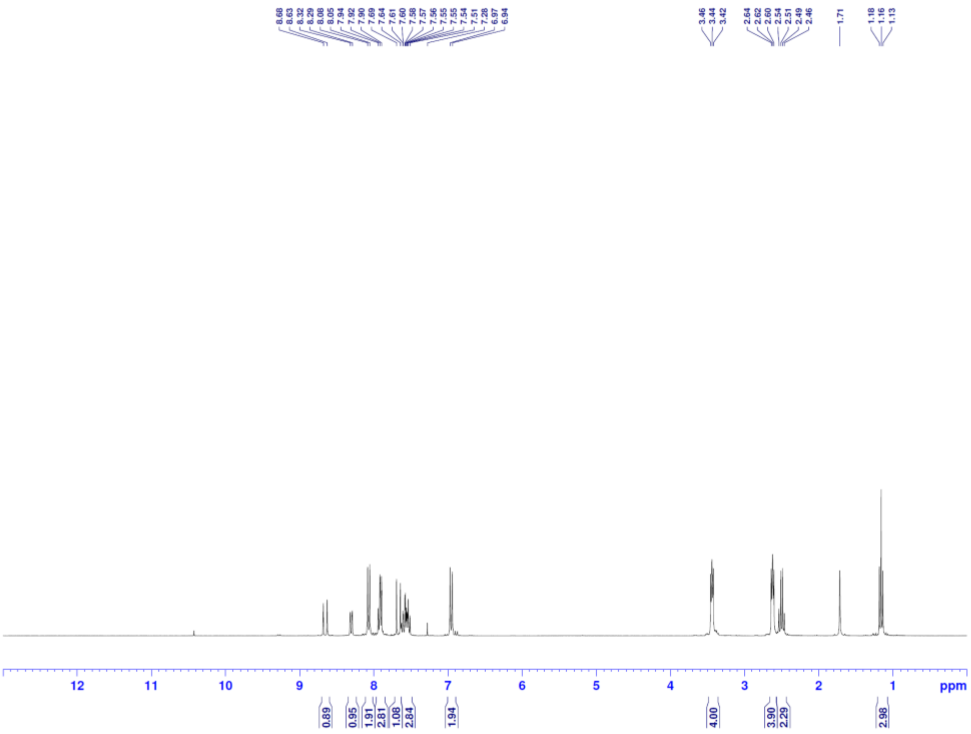

Figure S4. ^{1}H-NMR spectra of the compound **2b**

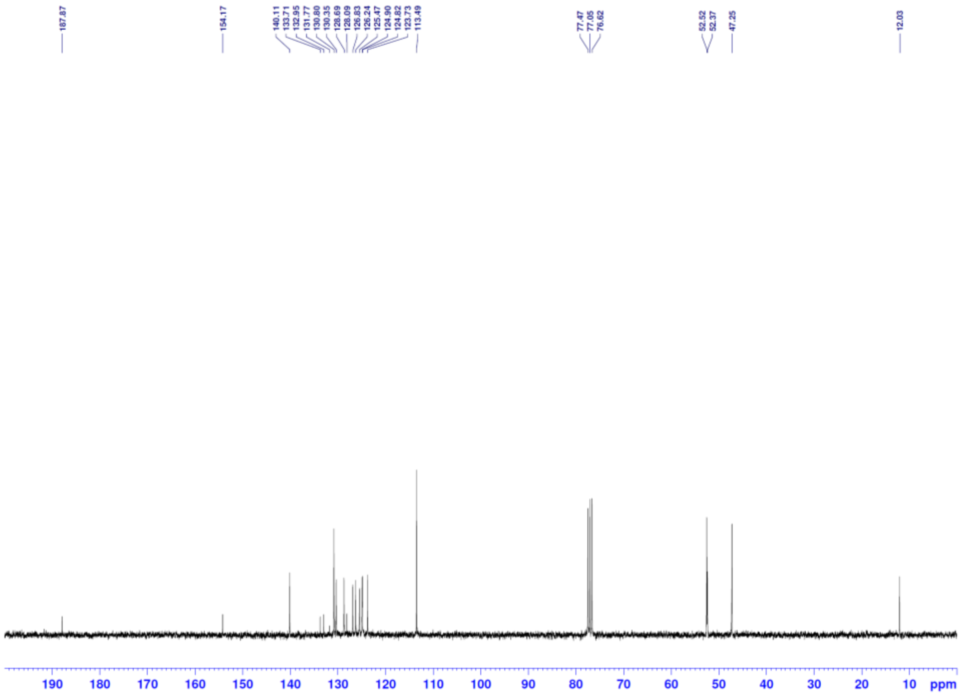

Figure S5. ^{13}C-NMR spectra of the compound **2b**

Formula Predictor Report - NK1-2_128.lcd Page 1 of 1

Data File: C:\LabSolutions\Data\Analiz\derya\NK1-2_128.lcd

Elmt	Val.	Min	Max	Elmt	Val.	Min	Max	Elmt	Val.	Min	Max	Elmt	Val.	Min	Max	Use Adduct
H	1	9	40	O	2	1	4	S	2	0	0	Ru	2	0	0	H
C	4	10	40	F	1	0	0	Cl	1	0	0	Pd	2	0	0	
N	3	1	5	P	3	0	0	Br	1	0	0	I	3	0	0	

Error Margin (ppm):	5	DBE Range:	5.0 - 25.0	Electron Ions:	both
HC Ratio:	unlimited	Apply N Rule:	yes	Use MSn Info:	yes
Max Isotopes:	3	Isotope RI (%):	1.00	Isotope Res:	9000
MSn Iso RI (%):	10.00	MSn Logic Mode:	AND	Max Results:	150

Event#: 1 MS(E+) Ret. Time : 2.627 Scan# : 395

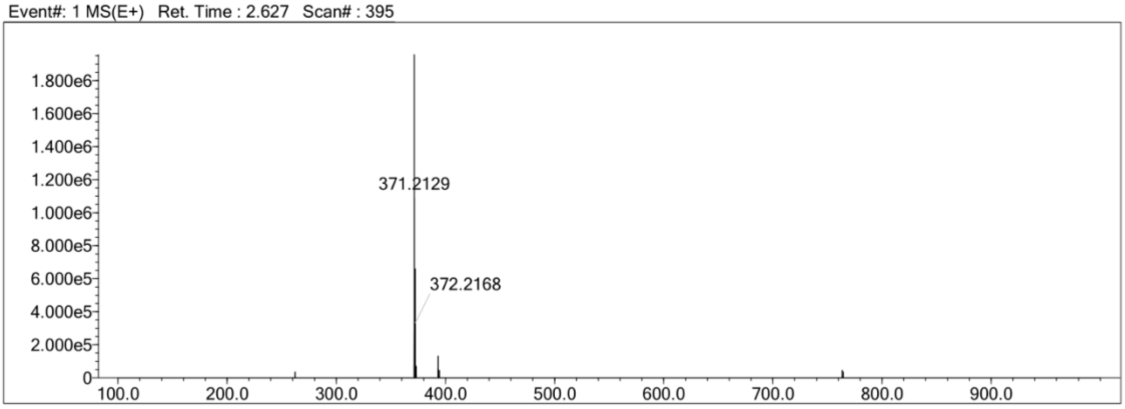

Measured region for 371.2129 m/z

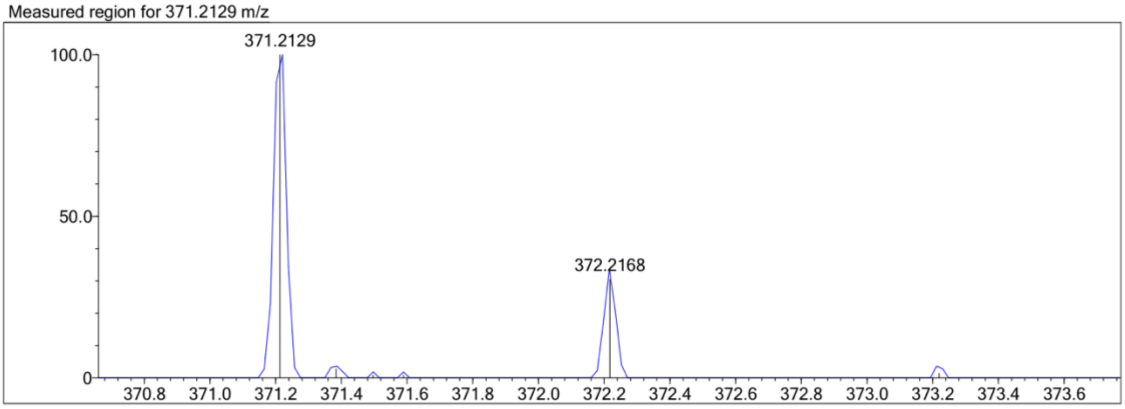

C25 H26 N2 O [M+H]+ : Predicted region for 371.2118 m/z

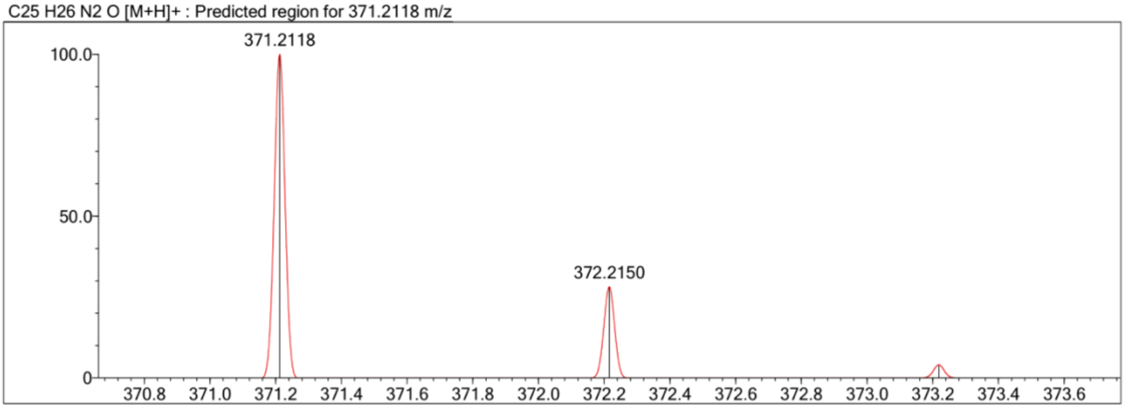

Rank	Score	Formula (M)	Ion	Meas. m/z	Pred. m/z	Df. (mDa)	Df. (ppm)	Iso	DBE
1	83.56	C25 H26 N2 O	[M+H]+	371.2129	371.2118	1.1	2.96	87.86	14.0

Figure S6. HRMS spectra of the compound **2b**

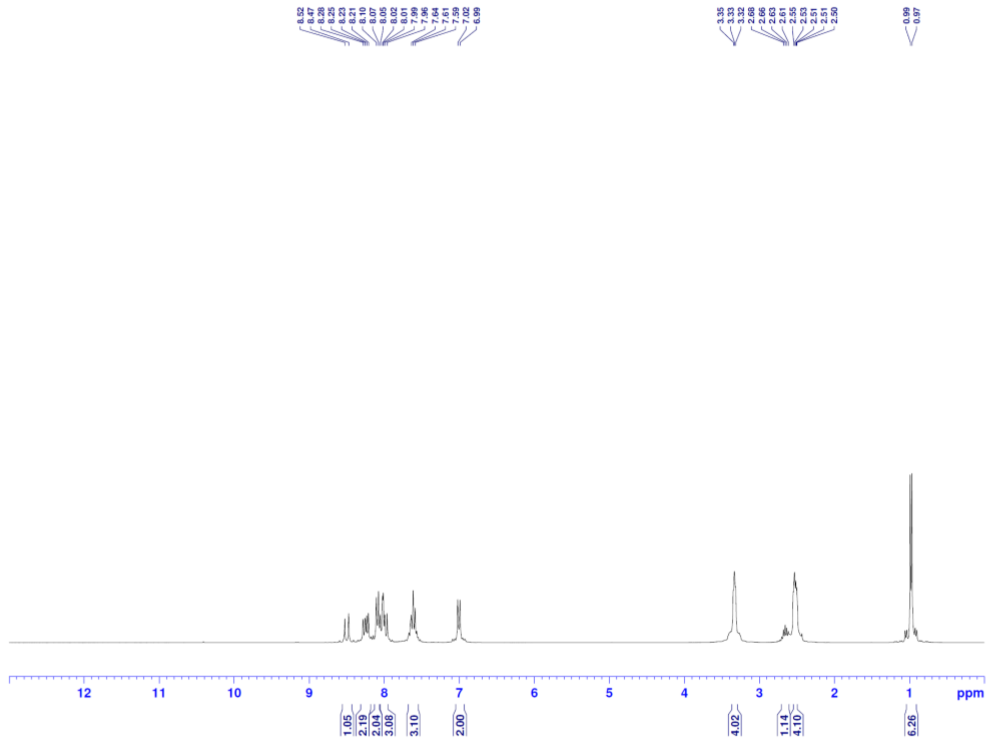

Figure S7. ^{1}H-NMR spectra of the compound **2c**

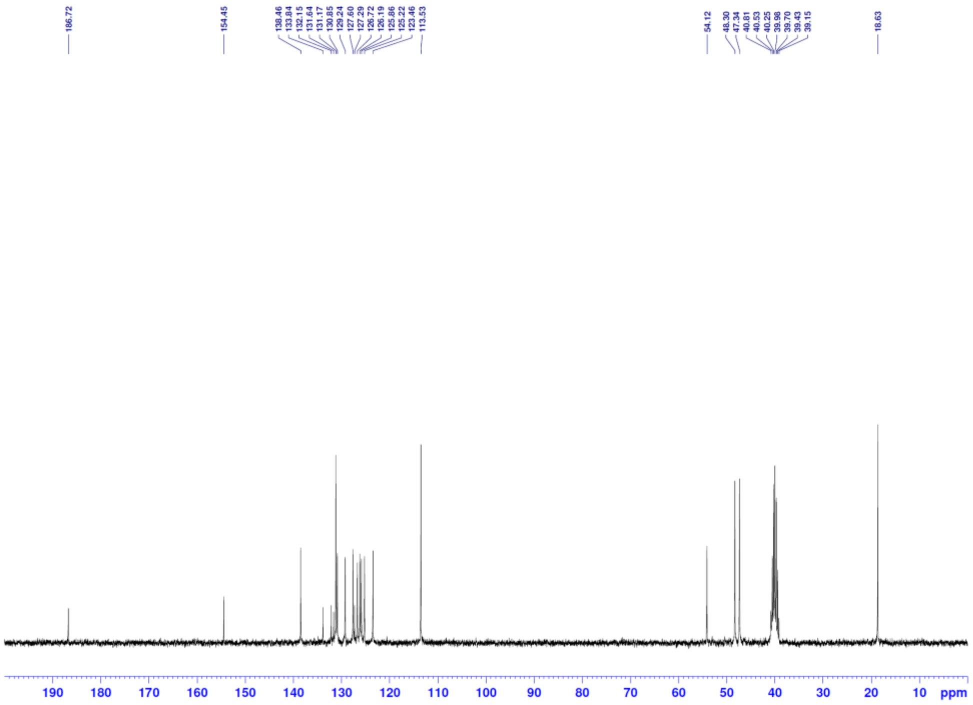

Figure S8. ^{13}C-NMR spectra of the compound **2c**

Formula Predictor Report - NK1-3_129.lcd

Page 1 of 1

Data File: C:\LabSolutions\Data\Analiz\derya\NK1-3_129.lcd

Elmt	Val.	Min	Max	Elmt	Val.	Min	Max	Elmt	Val.	Min	Max	Elmt	Val.	Min	Max	Use Adduct
H	1	9	40	O	2	1	4	S	2	0	0	Ru	2	0	0	H
C	4	10	40	F	1	0	0	Cl	1	0	0	Pd	2	0	0	
N	3	1	5	P	3	0	0	Br	1	0	0	I	3	0	0	

Error Margin (ppm):	5	DBE Range:	5.0 - 25.0	Electron Ions:	both
HC Ratio:	unlimited	Apply N Rule:	yes	Use MSn Info:	yes
Max Isotopes:	3	Isotope RI (%):	1.00	Isotope Res:	9000
MSn Iso RI (%):	10.00	MSn Logic Mode:	AND	Max Results:	150

Event#: 1 MS(E+) Ret. Time : 2.827 Scan# : 425

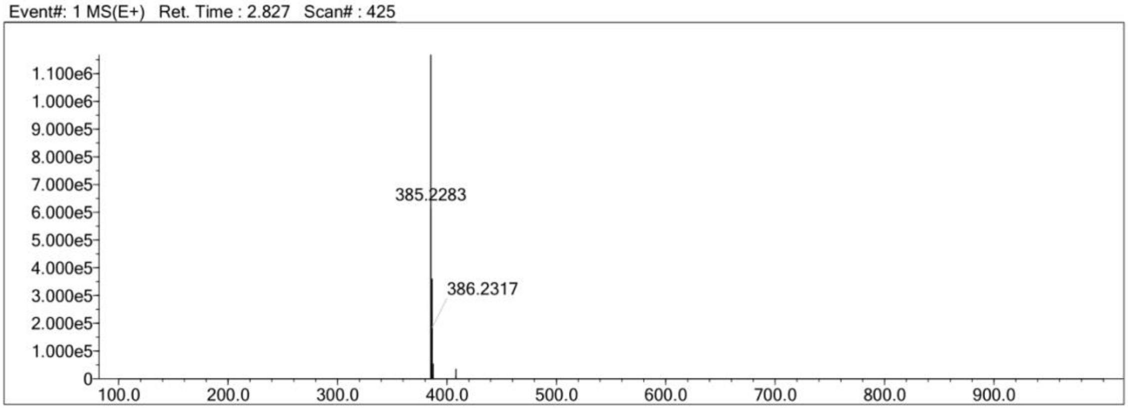

Measured region for 385.2283 m/z

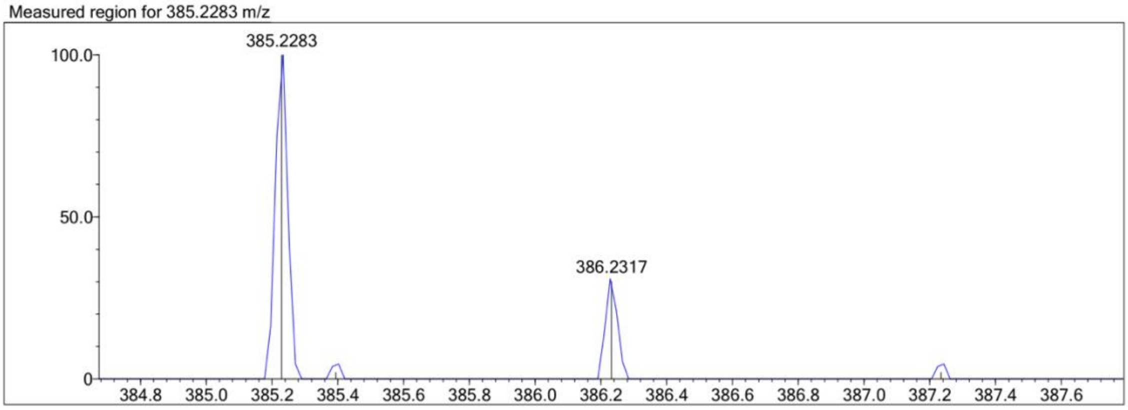

C26 H28 N2 O [M+H]+ : Predicted region for 385.2274 m/z

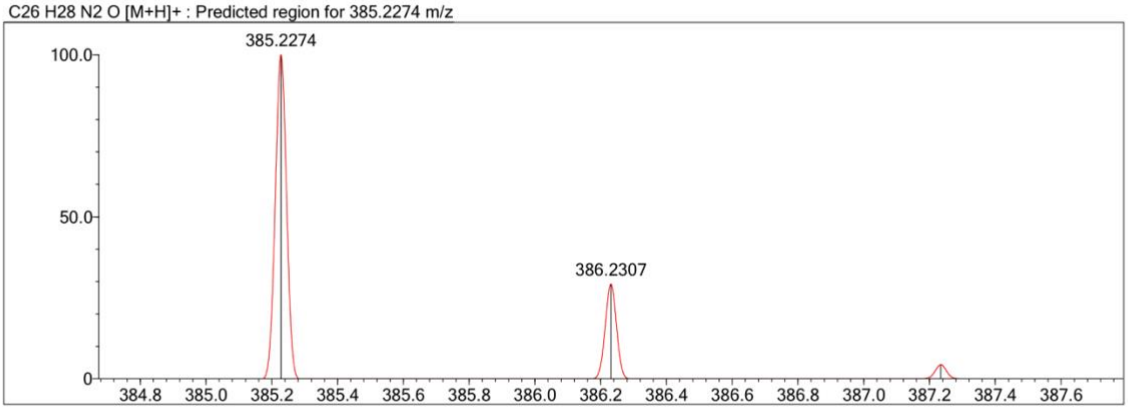

Rank	Score	Formula (M)	Ion	Meas. m/z	Pred. m/z	Df. (mDa)	Df. (ppm)	Iso	DBE
1	83.10	C26 H28 N2 O	[M+H]+	385.2283	385.2274	0.9	2.34	85.98	14.0

Figure S9. HRMS spectra of the compound **2c**

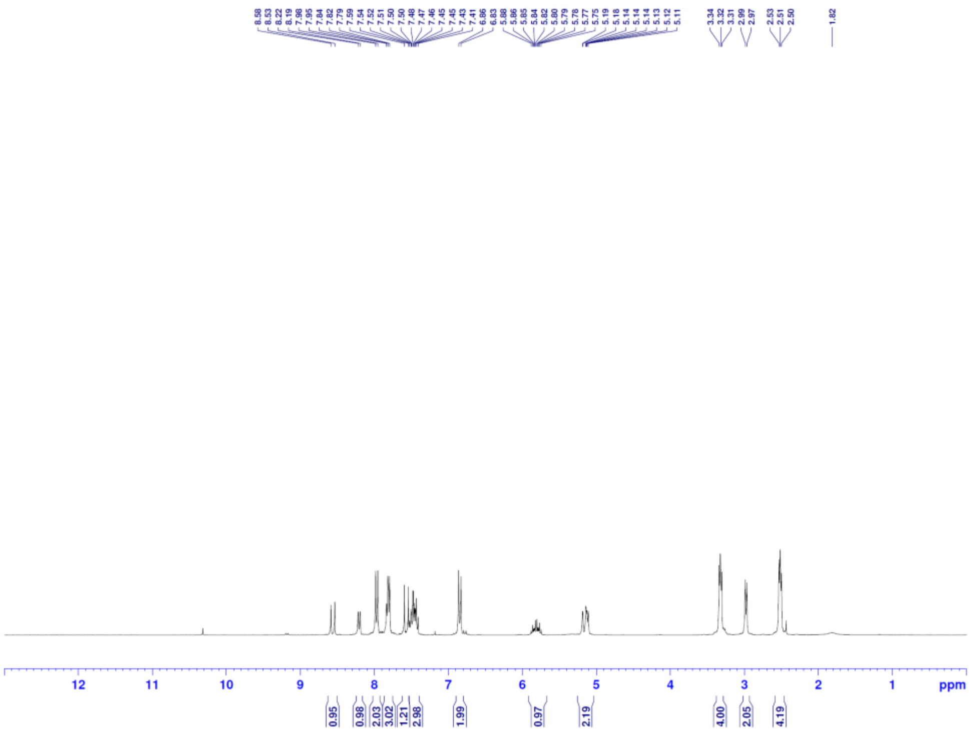

Figure S10. ^{1}H-NMR spectra of the compound **2d**

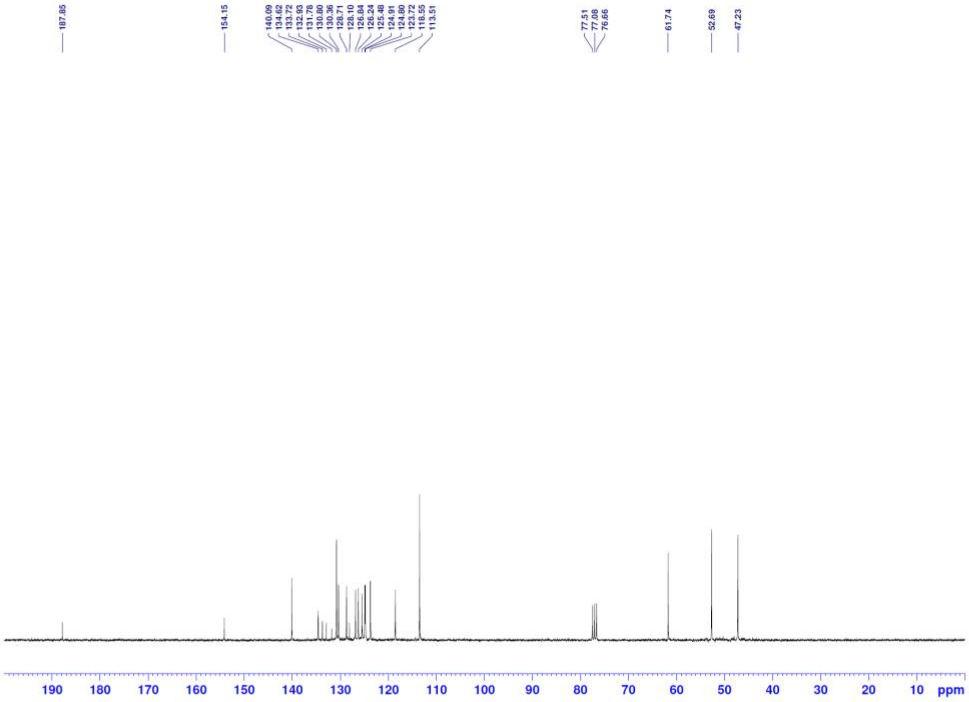

Figure S11. ^{13}C-NMR spectra of the compound **2d**

Formula Predictor Report - NK1-4_130.lcd

Page 1 of 1

Data File: C:\LabSolutions\Data\Analiz\derya\NK1-4_130.lcd

Elmt	Val.	Min	Max	Elmt	Val.	Min	Max	Elmt	Val.	Min	Max	Elmt	Val.	Min	Max	Use Adduct
H	1	9	40	O	2	1	4	S	2	0	0	Ru	2	0	0	H
C	4	10	40	F	1	0	0	Cl	1	0	0	Pd	2	0	0	
N	3	1	5	P	3	0	0	Br	1	0	0	I	3	0	0	

Error Margin (ppm):	5	DBE Range:	5.0 - 25.0	Electron Ions:	both
HC Ratio:	unlimited	Apply N Rule:	yes	Use MSn Info:	yes
Max Isotopes:	3	Isotope RI (%):	1.00	Isotope Res:	9000
MSn Iso RI (%):	10.00	MSn Logic Mode:	AND	Max Results:	150

Event#: 1 MS(E+) Ret. Time : 2.680 Scan# : 403

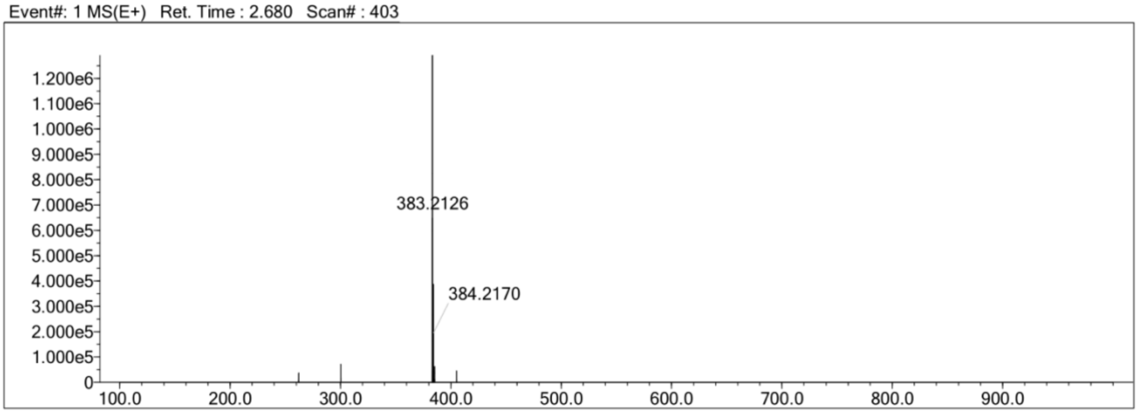

Measured region for 383.2126 m/z

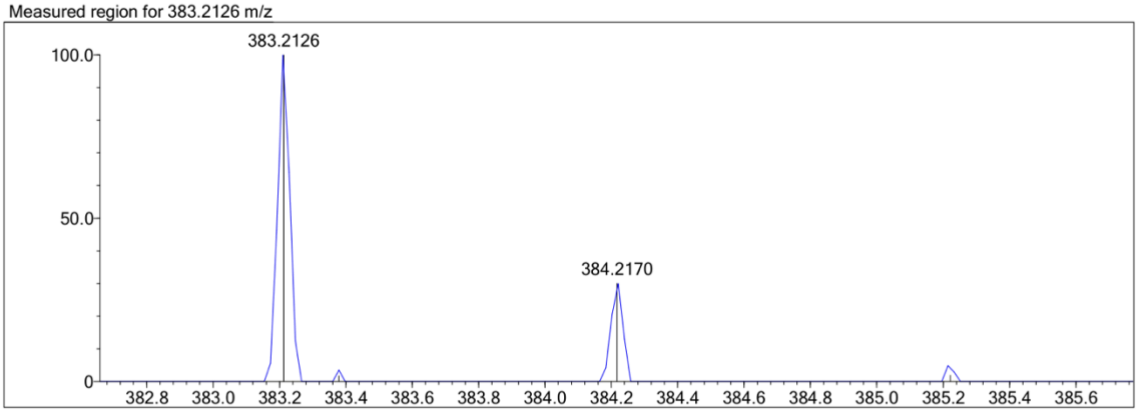

C26 H26 N2 O [M+H]+ : Predicted region for 383.2118 m/z

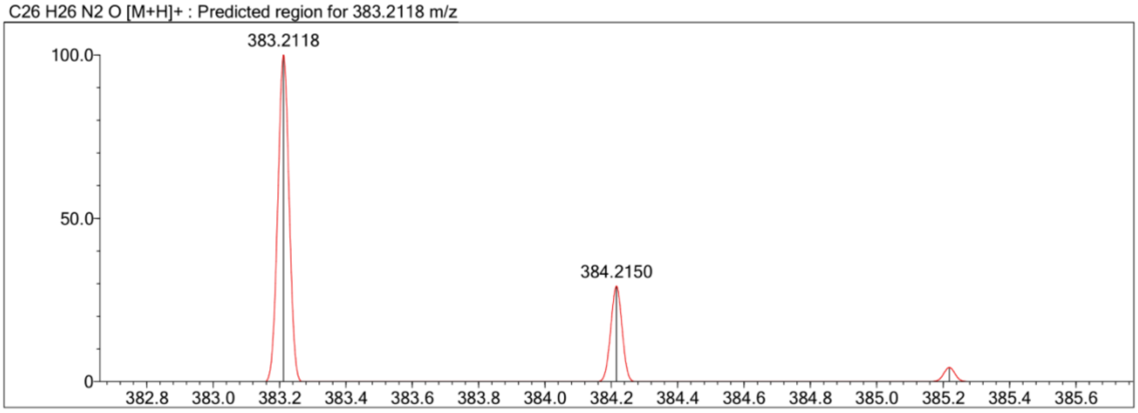

Rank	Score	Formula (M)	Ion	Meas. m/z	Pred. m/z	Df. (mDa)	Df. (ppm)	Iso	DBE
1	91.79	C26 H26 N2 O	[M+H]+	383.2126	383.2118	0.8	2.09	94.36	15.0

Figure S12. HRMS spectra of the compound **2d**

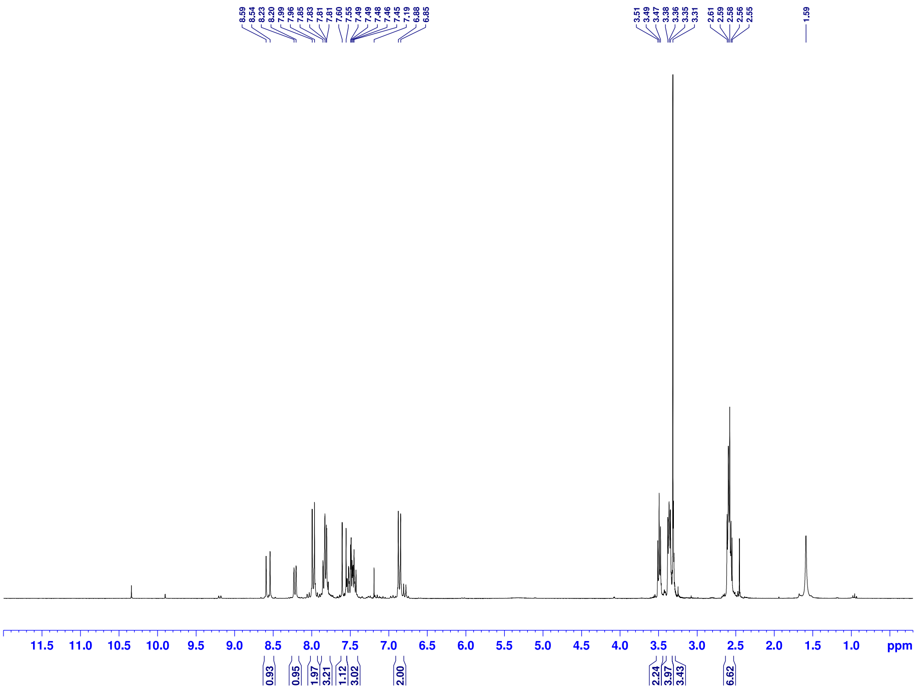

Figure S13. ^{1}H-NMR spectra of the compound **2e**

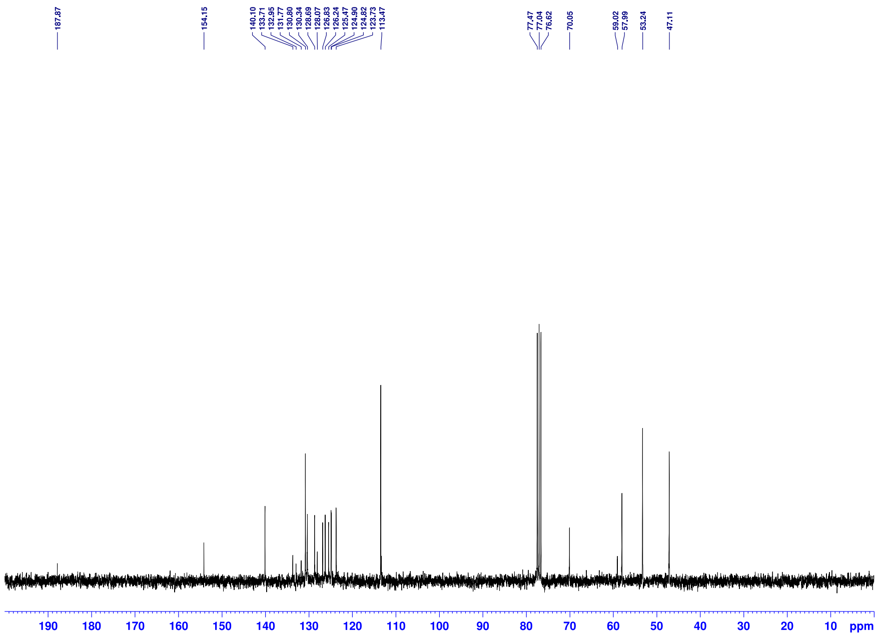

Figure S14. ^{13}C-NMR spectra of the compound **2e**

Data File: C:\LabSolutions\Data\Analiz\derya\NK1-5_131.lcd

Elmt	Val.	Min	Max	Elmt	Val.	Min	Max	Elmt	Val.	Min	Max	Elmt	Val.	Min	Max	Use Adduct
H	1	9	40	O	2	1	4	S	2	0	0	Ru	2	0	0	H
C	4	10	40	F	1	0	0	Cl	1	0	0	Pd	2	0	0	
N	3	1	5	P	3	0	0	Br	1	0	0	I	3	0	0	

Error Margin (ppm): 5
HC Ratio: unlimited
Max Isotopes: 3
MSn Iso RI (%): 10.00

DBE Range: 5.0 - 25.0
Apply N Rule: yes
Isotope RI (%): 1.00
MSn Logic Mode: AND

Electron Ions: both
Use MSn Info: yes
Isotope Res: 9000
Max Results: 150

Event#: 1 MS(E+) Ret. Time : 2.813 Scan# : 423

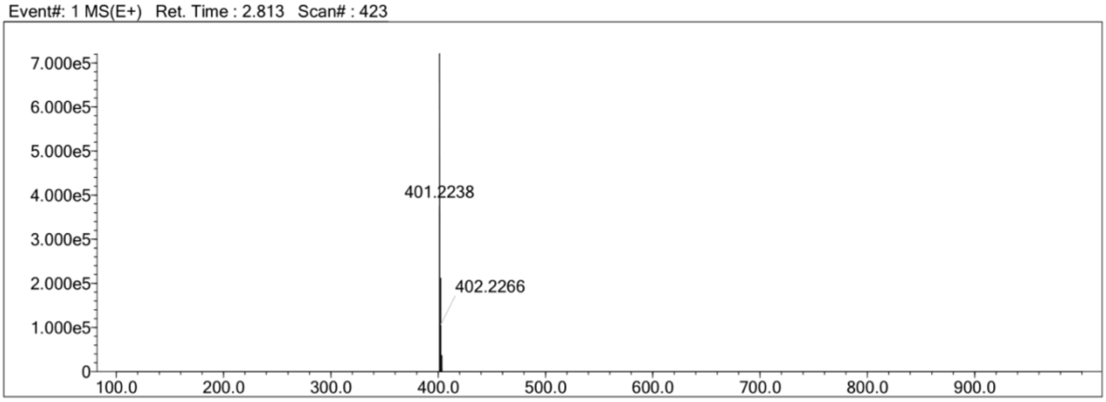

Measured region for 401.2238 m/z

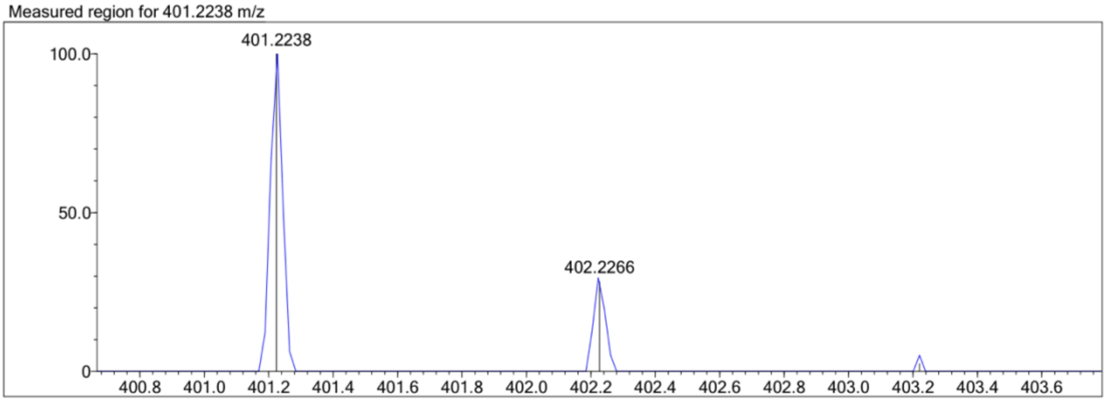

C26 H28 N2 O2 [M+H]+ : Predicted region for 401.2224 m/z

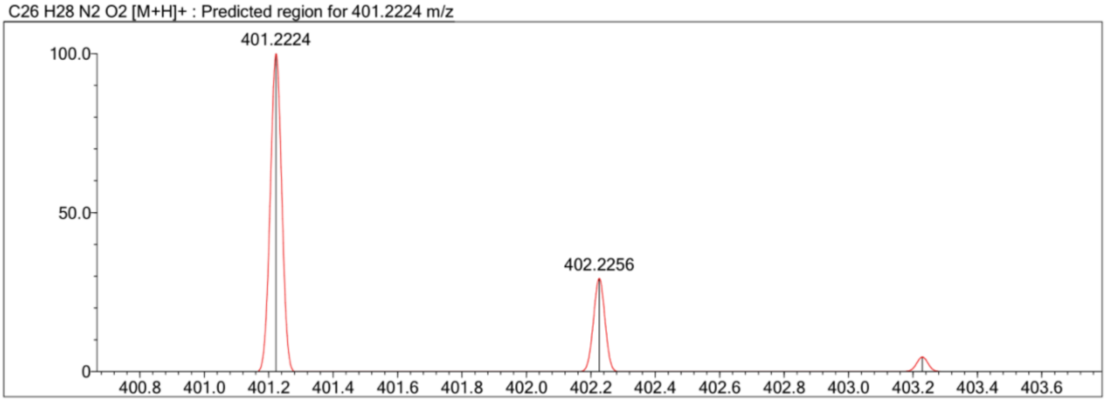

Rank	Score	Formula (M)	Ion	Meas. m/z	Pred. m/z	Df. (mDa)	Df. (ppm)	Iso	DBE
1	59.55	C26 H28 N2 O2	[M+H]+	401.2238	401.2224	1.4	3.49	63.50	14.0

Figure S15. HRMS spectra of the compound **2e**

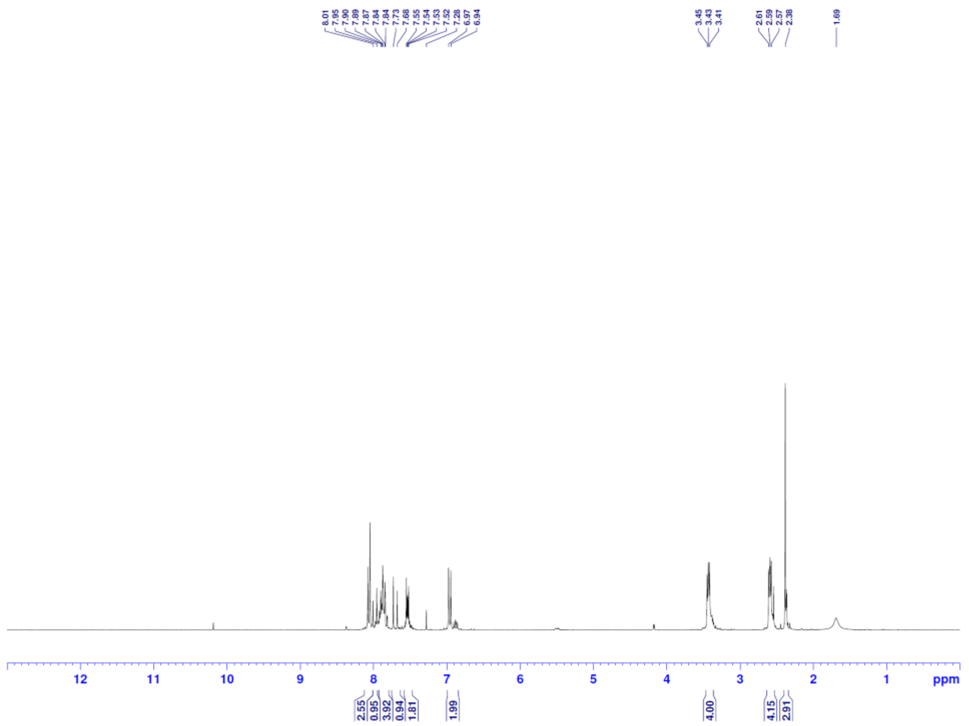

Figure S16. ^{1}H-NMR spectra of the compound **2f**

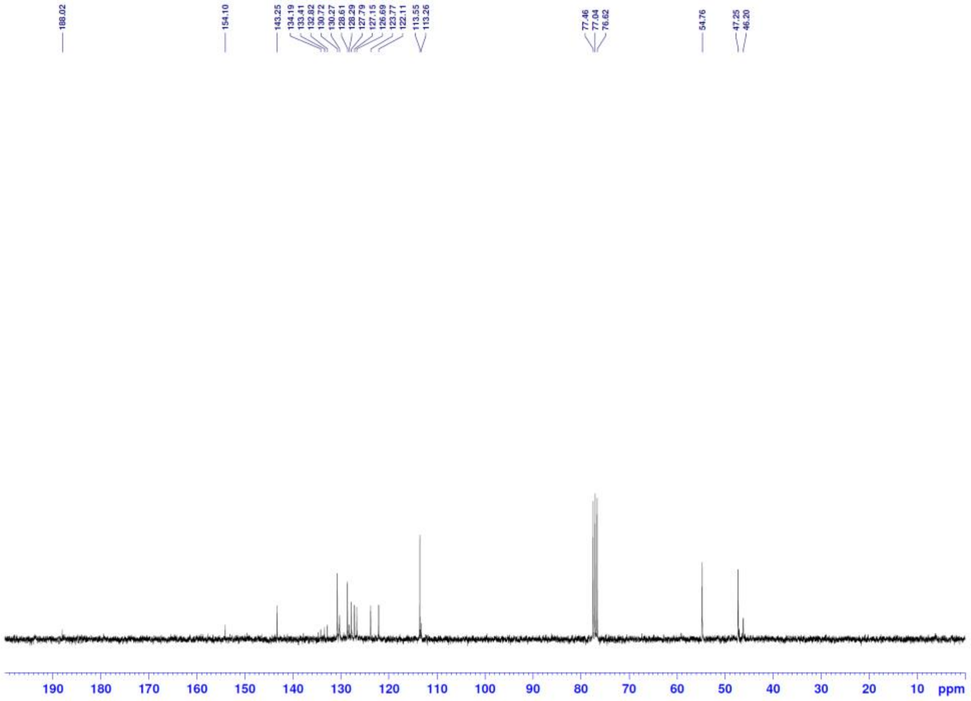

Figure S17. ^{13}C-NMR spectra of the compound **2f**

Data File: C:\LabSolutions\Data\Analiz\derya\NK2-1_134.lcd

Elmt	Val.	Min	Max	Elmt	Val.	Min	Max	Elmt	Val.	Min	Max	Elmt	Val.	Min	Max	Use Adduct
H	1	9	40	O	2	1	4	S	2	0	0	Ru	2	0	0	H
C	4	10	40	F	1	0	0	Cl	1	0	0	Pd	2	0	0	
N	3	1	5	P	3	0	0	Br	1	0	0	I	3	0	0	

Error Margin (ppm): 5
HC Ratio: unlimited
Max Isotopes: 3
MSn Iso RI (%): 10.00

DBE Range: 5.0 - 25.0
Apply N Rule: yes
Isotope RI (%): 1.00
MSn Logic Mode: AND

Electron Ions: both
Use MSn Info: yes
Isotope Res: 9000
Max Results: 150

Event#: 1 MS(E+) Ret. Time : 2.600 Scan# : 391

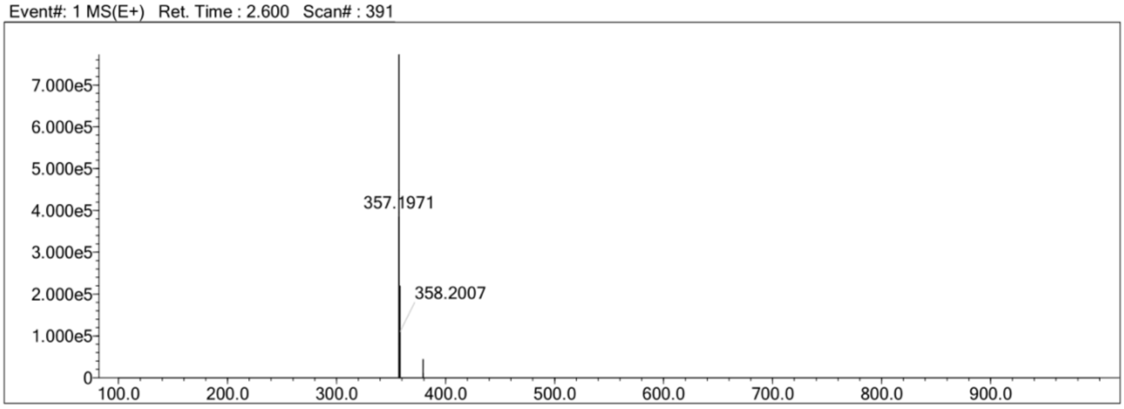

Measured region for 357.1971 m/z

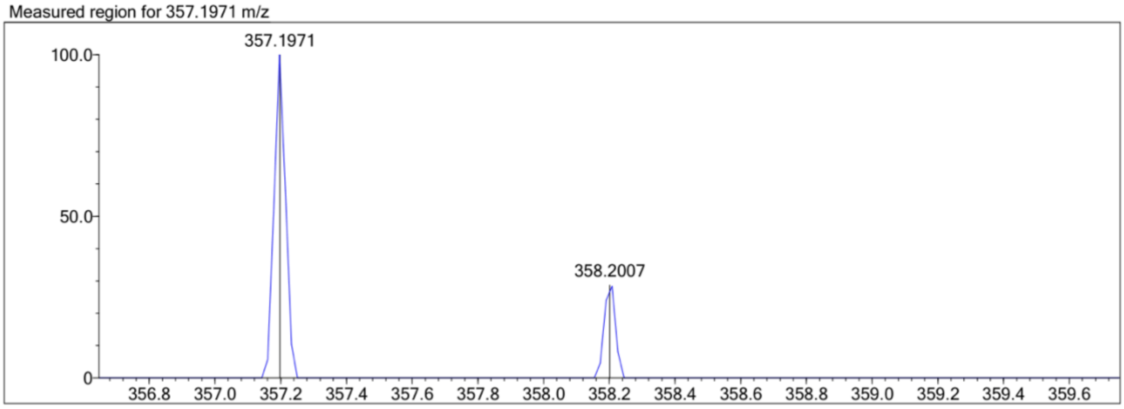

C24 H24 N2 O [M+H]+ : Predicted region for 357.1961 m/z

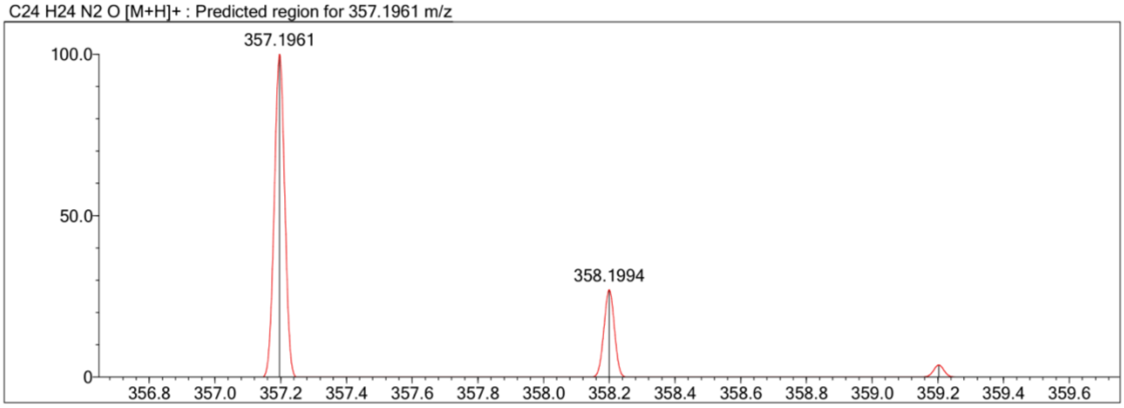

Rank	Score	Formula (M)	Ion	Meas. m/z	Pred. m/z	Df. (mDa)	Df. (ppm)	Iso	DBE
1	95.50	C24 H24 N2 O	[M+H]+	357.1971	357.1961	1.0	2.80	100.00	14.0

Figure S18. HRMS spectra of the compound **2f**

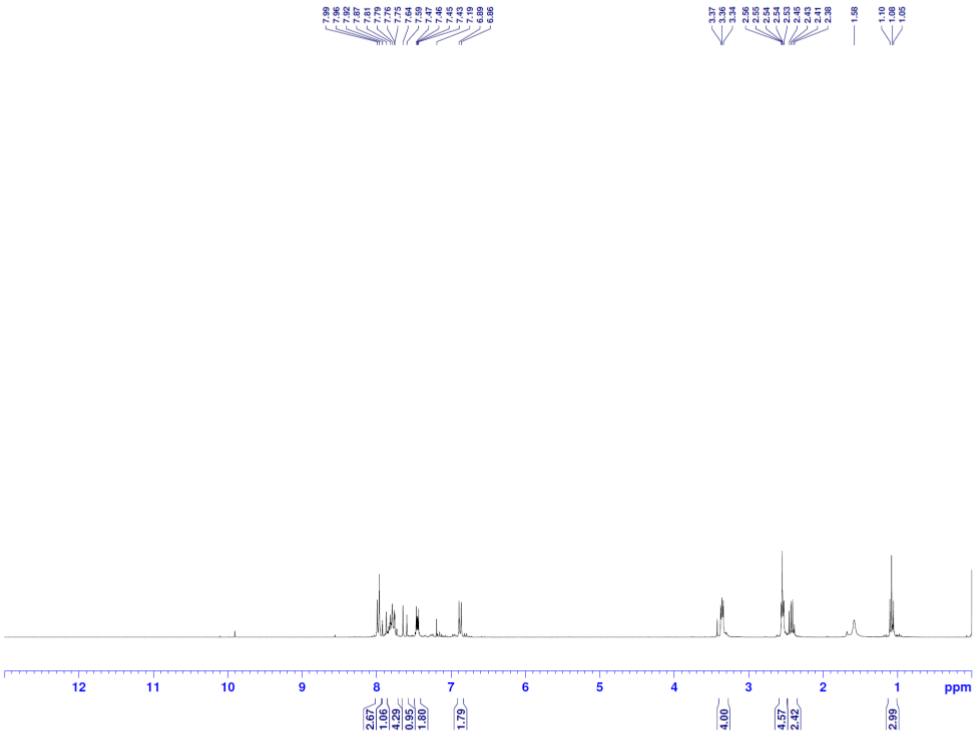

Figure S19. ^{1}H-NMR spectra of the compound **2g**

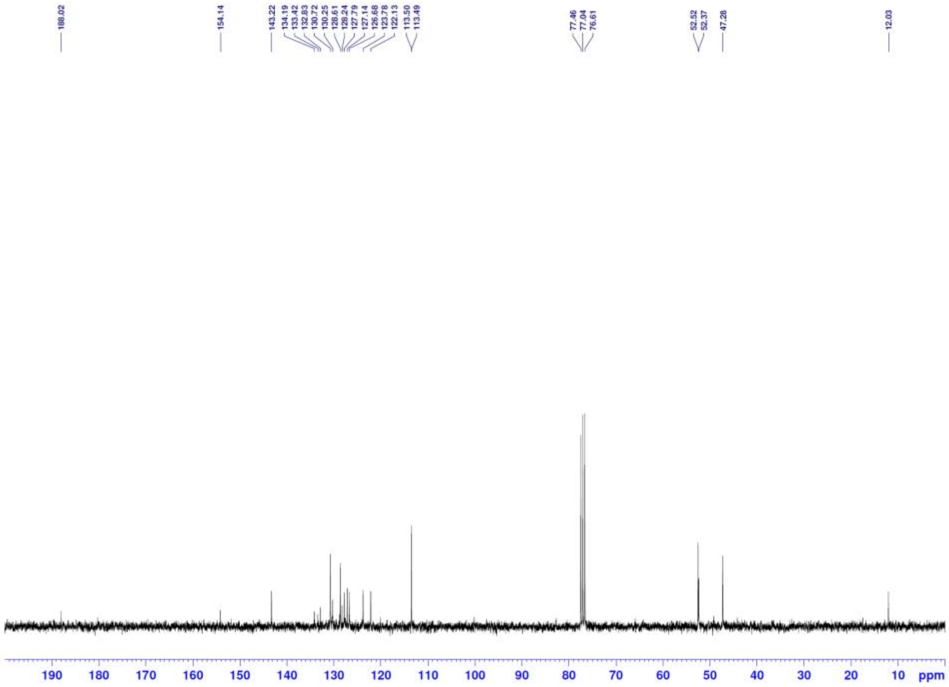

Figure S20. ^{13}C-NMR spectra of the compound **2g**

Formula Predictor Report - NK2-2_135.lcd Page 1 of 1

Data File: C:\LabSolutions\Data\Analiz\derya\NK2-2_135.lcd

Elmt	Val.	Min	Max	Elmt	Val.	Min	Max	Elmt	Val.	Min	Max	Elmt	Val.	Min	Max	Use Adduct
H	1	9	40	O	2	1	4	S	2	0	0	Ru	2	0	0	H
C	4	10	40	F	1	0	0	Cl	1	0	0	Pd	2	0	0	
N	3	1	5	P	3	0	0	Br	1	0	0	I	3	0	0	

Error Margin (ppm): 5
HC Ratio: unlimited
Max Isotopes: 3
MSn Iso RI (%): 10.00

DBE Range: 5.0 - 25.0
Apply N Rule: yes
Isotope RI (%): 1.00
MSn Logic Mode: AND

Electron Ions: both
Use MSn Info: yes
Isotope Res: 9000
Max Results: 150

Event#: 1 MS(E+) Ret. Time : 2.720 Scan# : 409

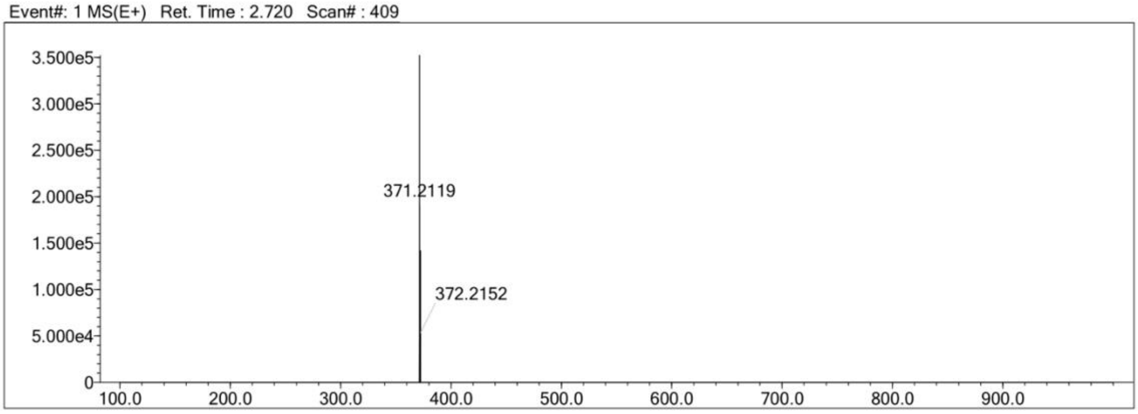

Measured region for 371.2119 m/z

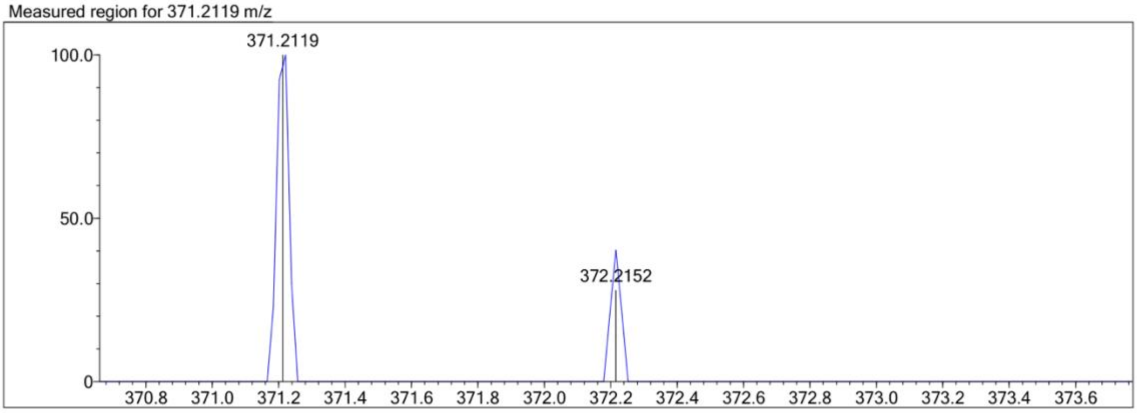

C25 H26 N2 O [M+H]+ : Predicted region for 371.2118 m/z

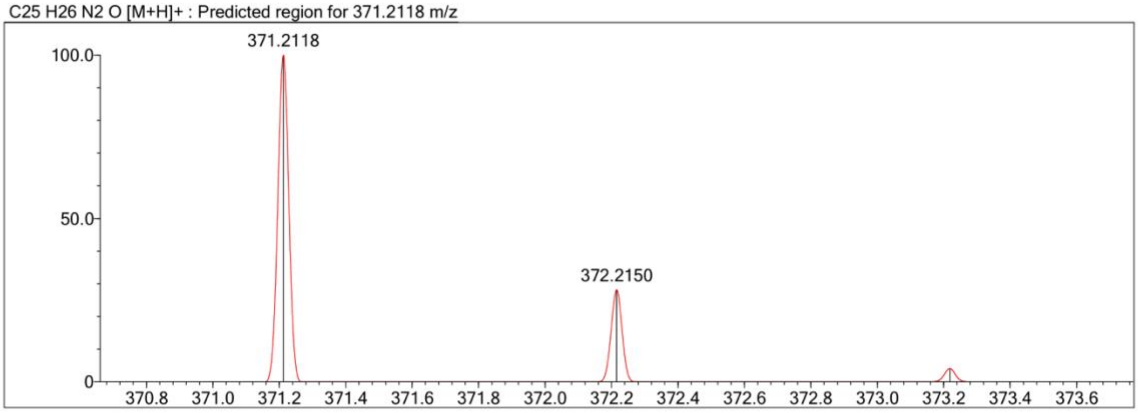

Rank	Score	Formula (M)	Ion	Meas. m/z	Pred. m/z	Df. (mDa)	Df. (ppm)	Iso	DBE
1	62.77	C25 H26 N2 O	[M+H]+	371.2119	371.2118	0.1	0.27	62.77	14.0

Figure S21. HRMS spectra of the compound **2g**

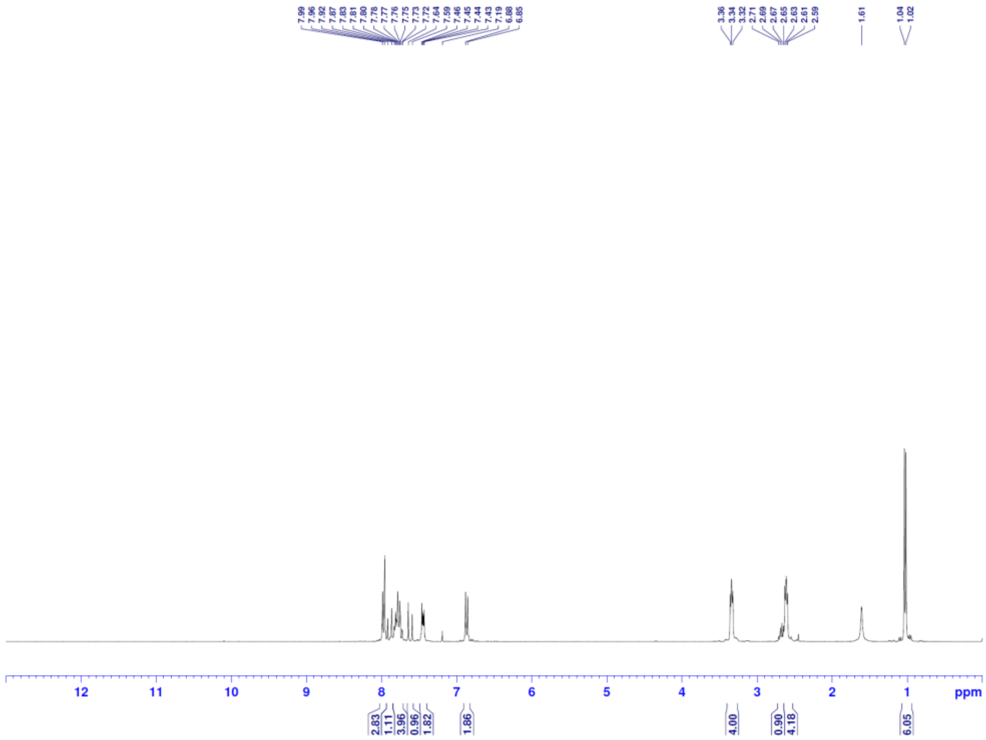

Figure S22. ^{1}H-NMR spectra of the compound **2h**

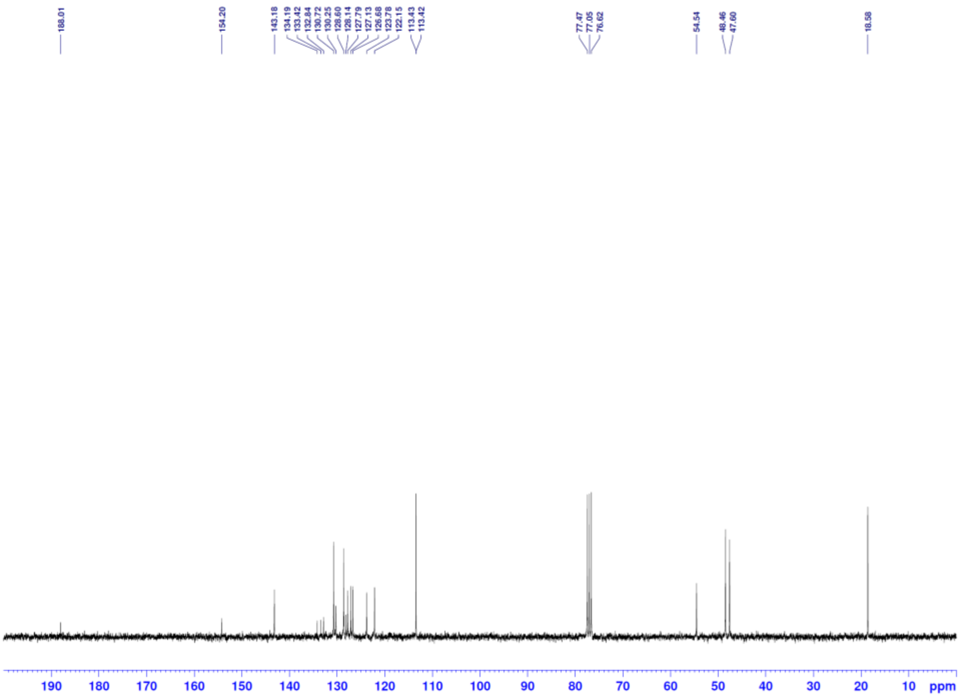

Figure S23. ^{13}C-NMR spectra of the compound **2h**

Formula Predictor Report - NK2-3_136.lcd

Data File: C:\LabSolutions\Data\Analiz\derya\NK2-3_136.lcd

Elmt	Val.	Min	Max	Elmt	Val.	Min	Max	Elmt	Val.	Min	Max	Elmt	Val.	Min	Max	Use Adduct
H	1	9	40	O	2	1	4	S	2	0	0	Ru	2	0	0	H
C	4	10	40	F	1	0	0	Cl	1	0	0	Pd	2	0	0	
N	3	1	5	P	3	0	0	Br	1	0	0	I	3	0	0	

Error Margin (ppm):	5	DBE Range:	5.0 - 25.0	Electron Ions:	both
HC Ratio:	unlimited	Apply N Rule:	yes	Use MSn Info:	yes
Max Isotopes:	3	Isotope RI (%):	1.00	Isotope Res:	9000
MSn Iso RI (%):	10.00	MSn Logic Mode:	AND	Max Results:	150

Event#: 1 MS(E+) Ret. Time : 2.880 Scan# : 433

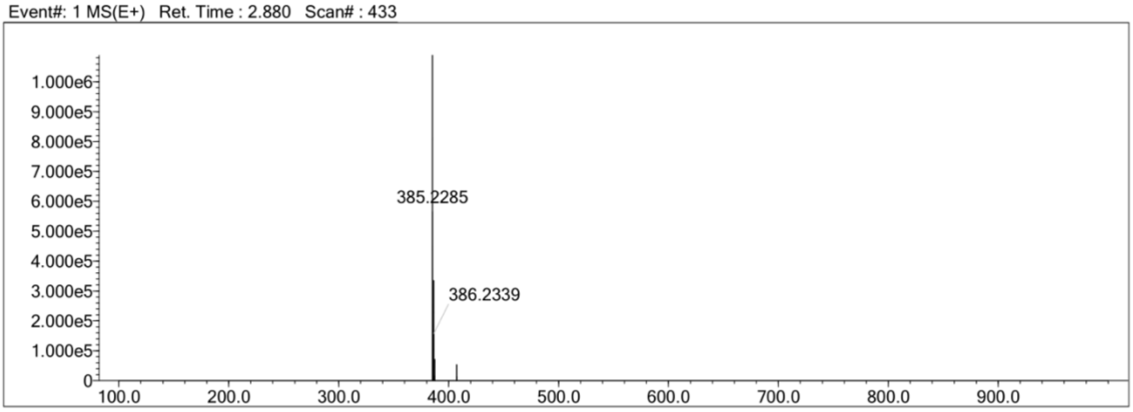

Measured region for 385.2285 m/z

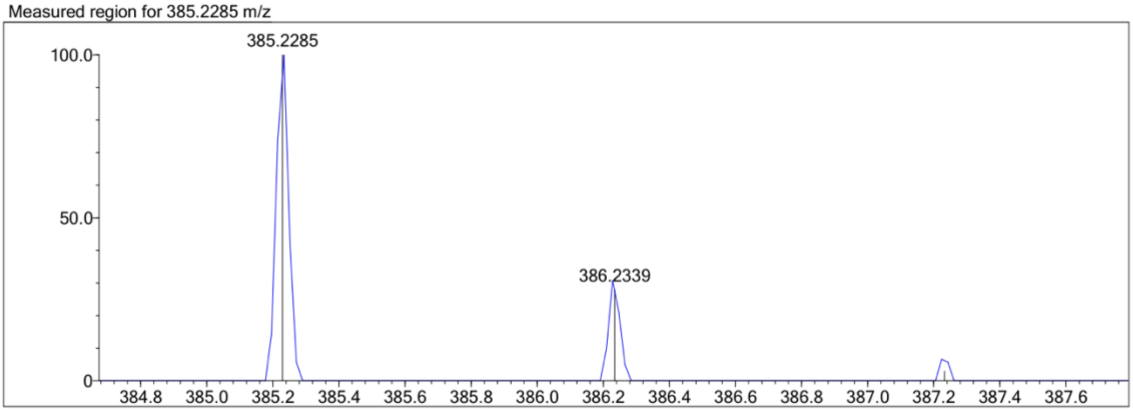

C26 H28 N2 O [M+H]+ : Predicted region for 385.2274 m/z

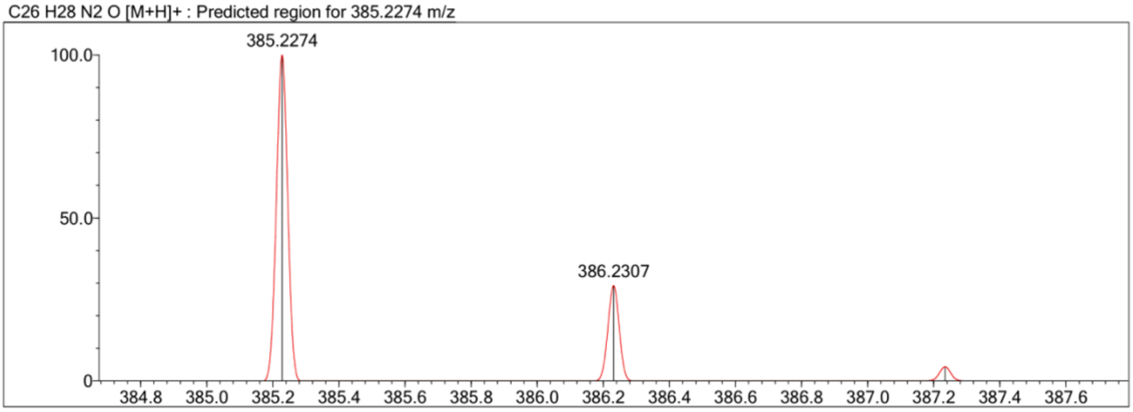

Rank	Score	Formula (M)	Ion	Meas. m/z	Pred. m/z	Df. (mDa)	Df. (ppm)	Iso	DBE
1	78.14	C26 H28 N2 O	[M+H]+	385.2285	385.2274	1.1	2.86	81.96	14.0

Figure S24. HRMS spectra of the compound **2h**

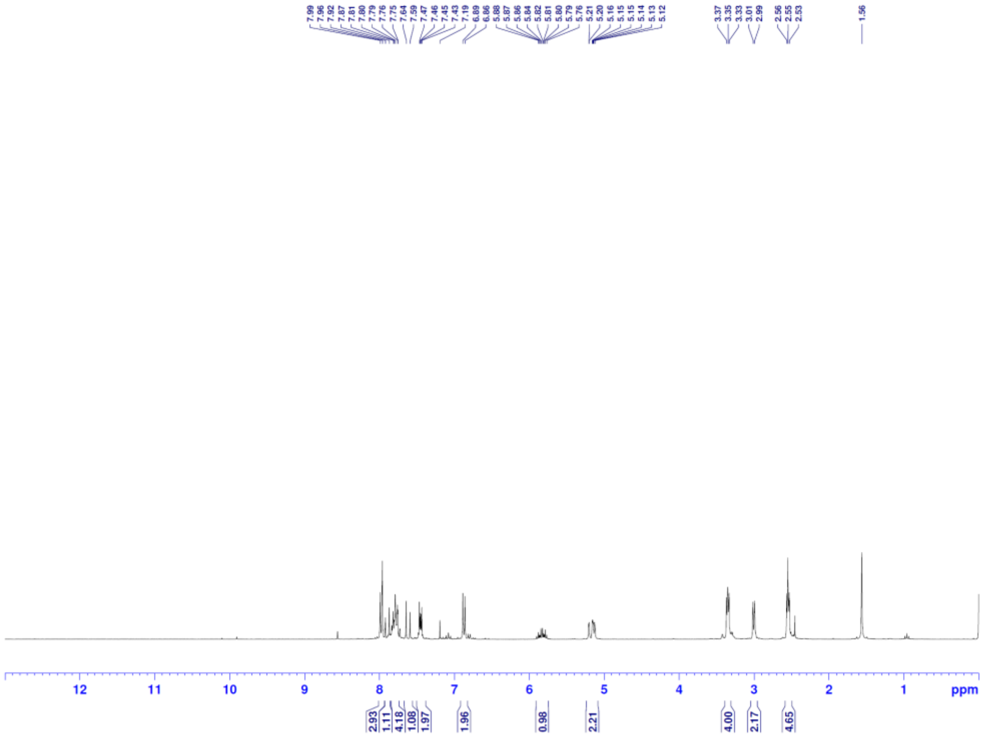

Figure S25. ^{1}H-NMR spectra of the compound **2i**

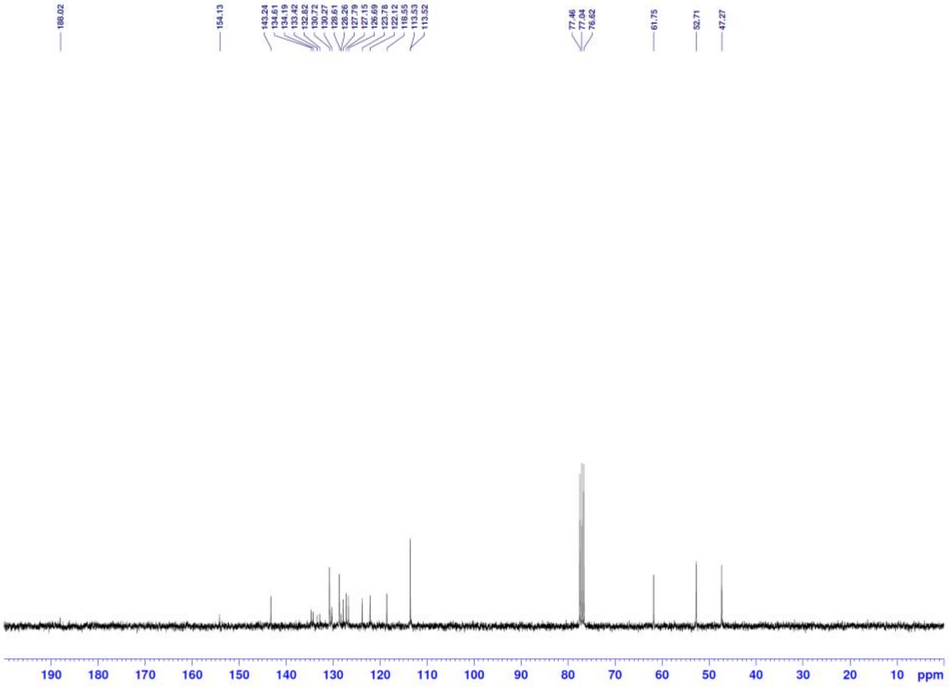

Figure S26. ^{13}C-NMR spectra of the compound **2i**

Data File: C:\LabSolutions\Data\Analiz\derya\NK2-4_137.lcd

Elmt	Val.	Min	Max	Elmt	Val.	Min	Max	Elmt	Val.	Min	Max	Elmt	Val.	Min	Max	Use Adduct
H	1	9	40	O	2	1	4	S	2	0	0	Ru	2	0	0	H
C	4	10	40	F	1	0	0	Cl	1	0	0	Pd	2	0	0	
N	3	1	5	P	3	0	0	Br	1	0	0	I	3	0	0	

Error Margin (ppm):	5	DBE Range:	5.0 - 25.0	Electron Ions:	both
HC Ratio:	unlimited	Apply N Rule:	yes	Use MSn Info:	yes
Max Isotopes:	3	Isotope RI (%):	1.00	Isotope Res:	9000
MSn Iso RI (%):	10.00	MSn Logic Mode:	AND	Max Results:	150

Event#: 1 MS(E+) Ret. Time : 2.867 Scan# : 431

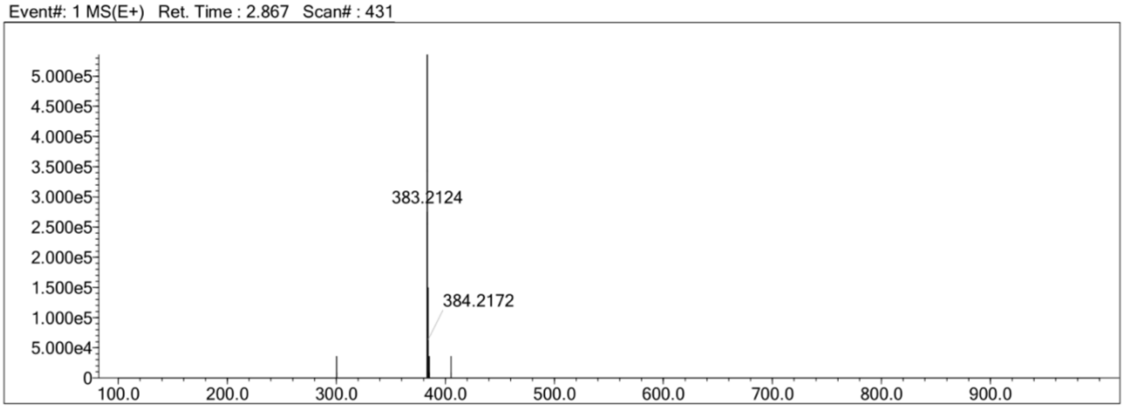

Measured region for 383.2124 m/z

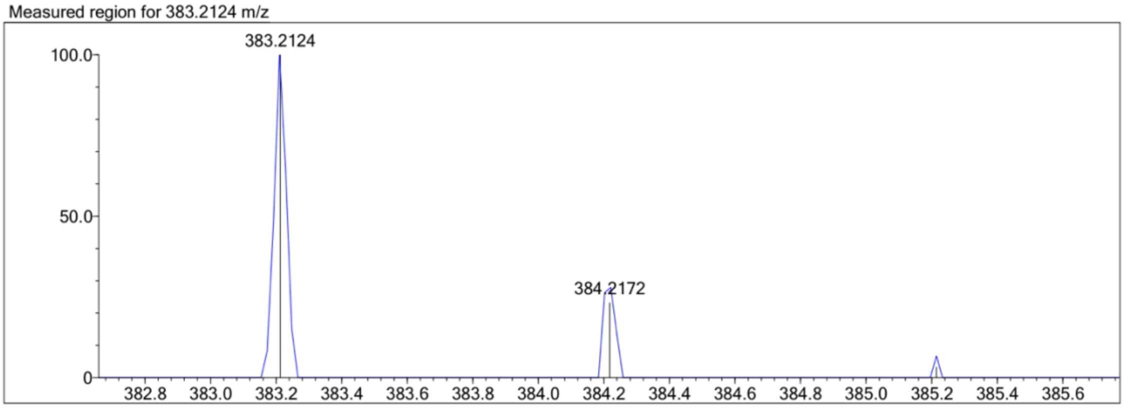

C26 H26 N2 O [M+H]+ : Predicted region for 383.2118 m/z

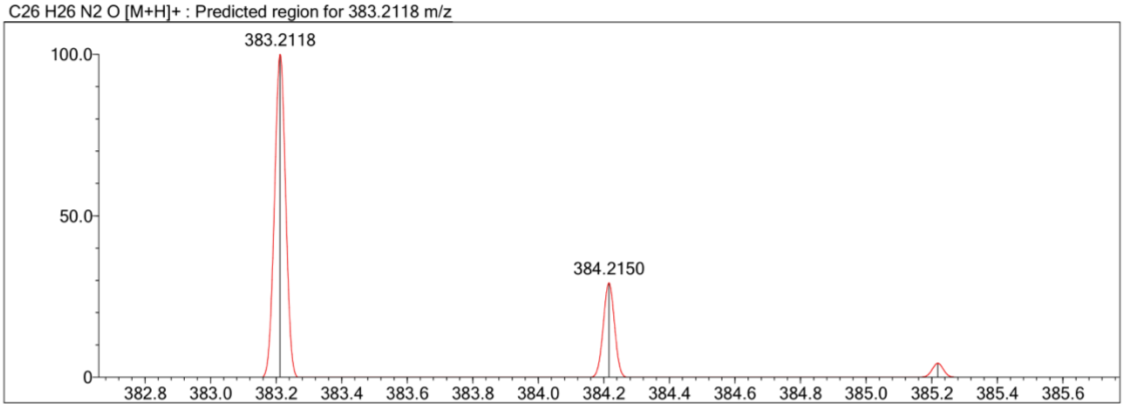

Rank	Score	Formula (M)	Ion	Meas. m/z	Pred. m/z	Df. (mDa)	Df. (ppm)	Iso	DBE
1	87.63	C26 H26 N2 O	[M+H]+	383.2124	383.2118	0.6	1.57	88.89	15.0

Figure S27. HRMS spectra of the compound **2i**

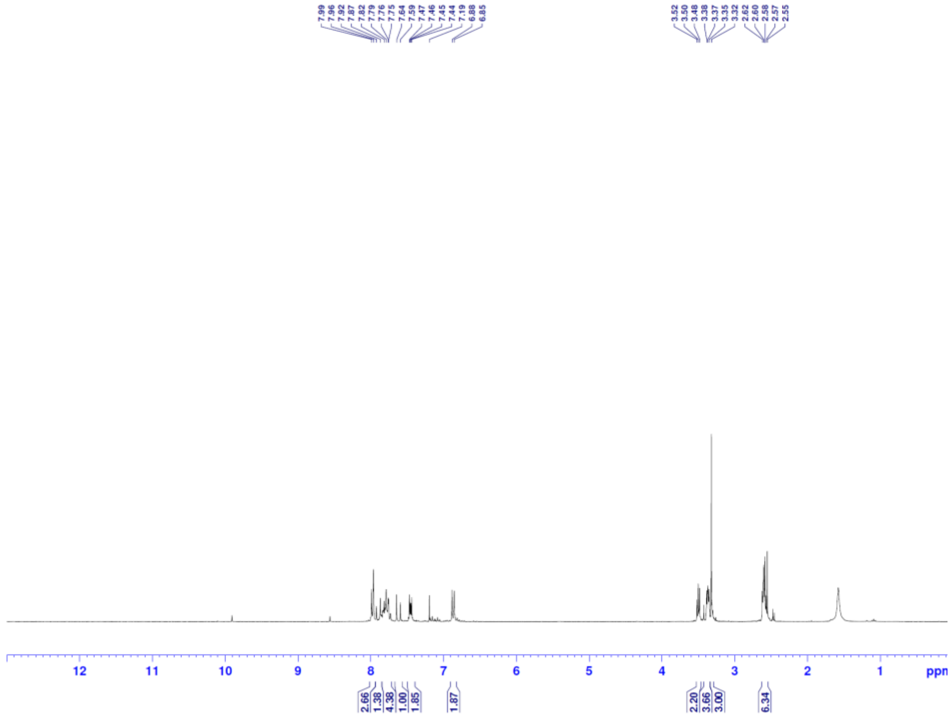

Figure S28. ^{1}H-NMR spectra of the compound **2j**

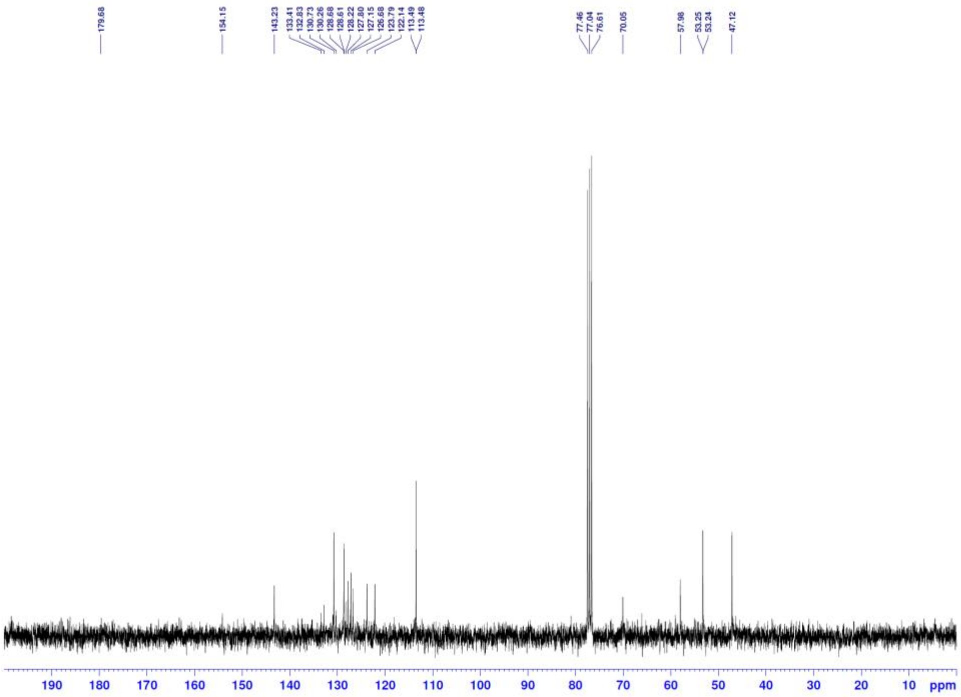

Figure S29. ^{13}C-NMR spectra of the compound **2j**

Data File: C:\LabSolutions\Data\Analiz\derya\NK2-5_138.lcd

Elmt	Val.	Min	Max
H	1	9	40
C	4	10	40
N	3	1	5

Elmt	Val.	Min	Max
O	2	1	4
F	1	0	0
P	3	0	0

Elmt	Val.	Min	Max
S	2	0	0
Cl	1	0	0
Br	1	0	0

Elmt	Val.	Min	Max
Ru	2	0	0
Pd	2	0	0
I	3	0	0

Use Adduct
H

Error Margin (ppm): 5
HC Ratio: unlimited
Max Isotopes: 3
MSn Iso RI (%): 10.00

DBE Range: 5.0 - 25.0
Apply N Rule: yes
Isotope RI (%): 1.00
MSn Logic Mode: AND

Electron Ions: both
Use MSn Info: yes
Isotope Res: 9000
Max Results: 150

Event#: 1 MS(E+) Ret. Time : 2.773 Scan# : 417

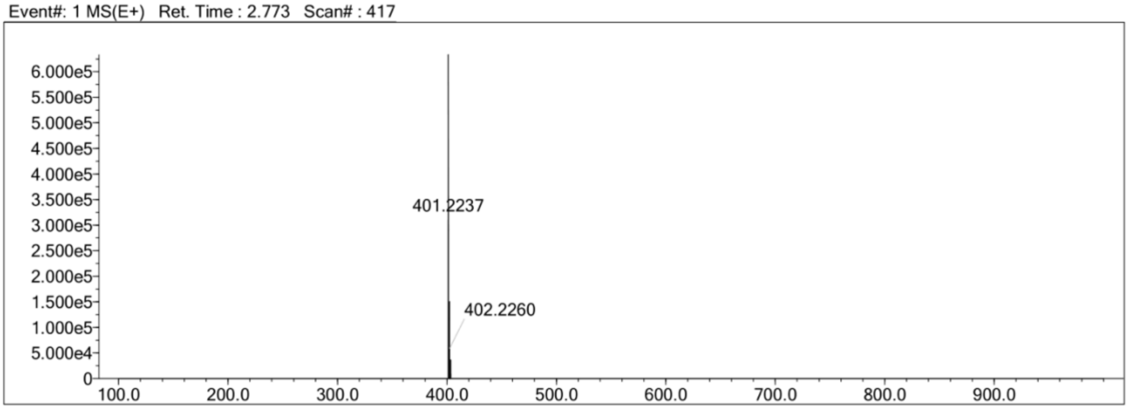

Measured region for 401.2237 m/z

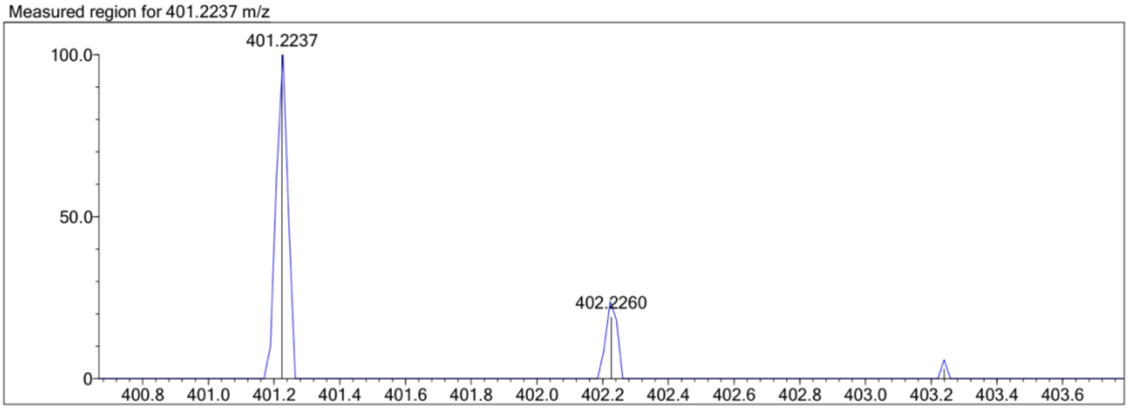

C26 H28 N2 O2 [M+H]+ : Predicted region for 401.2224 m/z

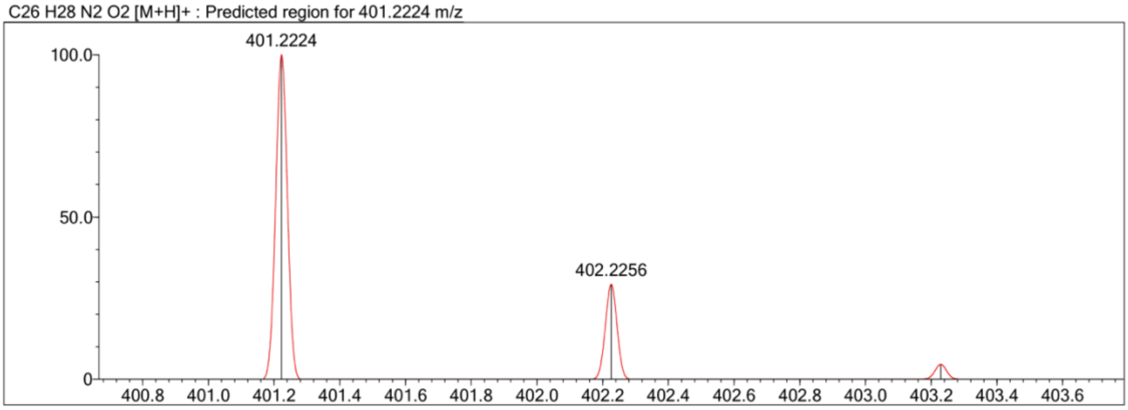

Rank	Score	Formula (M)	Ion	Meas. m/z	Pred. m/z	Df. (mDa)	Df. (ppm)	Iso	DBE
1	51.37	C26 H28 N2 O2	[M+H]+	401.2237	401.2224	1.3	3.24	54.41	14.0

Figure S30. HRMS spectra of the compound **2j**
